# Supplementary material for: Photocatalytic aerobic oxidation of C(sp3)-H bonds
Source: Nat Commun. 2024 Jan 15;15:537. doi: 10.1038/s41467-024-44833-y (PMC10789790; doi:10.1038/s41467-024-44833-y)
Supplement: Supplementary file 1 — Supplementary Information [file 41467_2024_44833_MOESM1_ESM.pdf]

Supplementary Information for

Photocatalytic aerobic oxidation of C(sp^3)-H bonds

Lei Zhang[1†], Run-Han Li[1†], Xiao-Xin Li[1*], Shengyao Wang[2], Jiang Liu[1], Xiao-Xuan Hong[1], Long-Zhang Dong[1], Shun-Li Li[1], and Ya-Qian Lan[1*]

[1]School of Chemistry, South China Normal University, Guangzhou 510631, P. R. China.

[2] College of Science, Huazhong Agricultural University, Wuhan, 430070, P. R. China.

†These authors contributed equally to this work.

Corresponding Author:

*E-mail: xxli@m.scnu.edu.cn; yqlan@m.scnu.edu.cn.

S1. Materials and measurements

All starting materials, reagents and solvents used in experiments were commercially available, high-grade purity materials and used without further purification. 3,5-dibromoaniline ($C_6H_3NH_2Br_2$), propanoic acid (C_2H_5COOH), 1,6,7,12-Tetrachloroperylene Tetracarboxylic Acid Dianhydride ($C_{24}H_4Cl_4O_6$), ammonium cerium nitrate ($Ce(NH_4)_2(NO_3)_6$), glycine (NH_2CH_2COOH) were purchased from Aladdin. Sodium chloride (NaCl), Benzoic acid (BA, C_6H_5COOH), 1-naphthoic acid (NA, $C_{10}H_7COOH$), 9-anthroic acid (9-AC, $C_{14}H_9COOH$) were purchased from Shanghai Haohong Scientific Co. Ltd. Toluene was purchased from Guangzhou Chemical Reagent Factory. Ethylbenzene was purchased from Energy Chemical. Phenethyl ethanol, anisole sulfide, benzylamine and their derivatives were purchased from Adamas Reagent Co. Ltd.

Supplementary Table 1. Crystal data and structure refinement of CeBTTD (-B, -N and -A), respectively.

	CeBTTD-B	CeBTTD-N	CeBTTD-A
Empirical formula	$C_{27}H_{14}CeCl_2NO_{10}$	$C_{32}H_{16}CeCl_2NO_{10}$	$C_{38}H_{23}CeCl_2N_2O_{10}$
Formula weight	723.41	785.48	878.60
Temperature/K	150	150	150
Crystal system	Monoclinic	Monoclinic	Orthorhombic
Space group	*C2/c*	*C2/c*	*Pnma*
a/Å	41.87(2)	41.778(16)	16.7769(4)
b/Å	13.940(7)	13.452(5)	42.5051(9)
c/Å	14.711(8)	15.098(6)	11.4038(3)
α/°	90	90	90
β/°	104.626(16)	99.819(13)	90
γ/°	90	90	90
Volume/Å^3	8308(2)	8361(5)	8132.1(3)
Z	8	8	8
ρ_{calc} g/cm^3	1.157	1.248	1.435
μ/mm^{-1}	9.998	9.978	10.329
F(000)	2840.0	3096	3496.0
Reflections collected	10206	16611	40067
Independent reflections	3944[R_{int} = 0.0757, R_{sigma} = 0.0931]	4205[R_{int} = 0.0718, R_{sigma} = 0.0651]	8027 [R_{int} = 0.0884, R_{sigma} = 0.0769]
Data/restraints/parameters	3944/36/372	4205/355/463	8027/568/688
Goodness-of-fit on F^2	1.082	1.064	1.037
Final R indexes [I>=2σ (I)]	R_1 = 0.0824, wR_2 = 0.2301	R_1 =0.0813, wR_2 = 0.2182	R_1 = 0.0756, wR_2 = 0.2087
Final R indexes [all data]	R_1 = 0.1084, wR_2 = 0.2563	R_1 = 0.1038, wR_2 = 0.2458	R_1 = 0.1173, wR_2 = 0.2482
Largest diff. peak/hole / e Å^{-3}	1.09/-1.99	1.12/-1.20	0.83/-1.32

$^aR_1$=Σ||F_o|- |F_c||/Σ|F_o|. $^bwR_2$= |Σw (|F_o|^2-|F_c|^2)|/Σ|$w(F_o^2)^2$|$^{1/2}$

Supplementary Table 2. Selected bond lengths (Å) of CeBTTD-B.

Atom1	Atom2	Length/ Å	Atom1	Atom2	Length/ Å
Ce1	$O6^{1}$	2.478	Ce1	O4	2.561
Ce1	$O6^{2}$	2.724	Ce1	O3	2.573
Ce1	O1	2.464	Ce1	O1w	2.497
Ce1	$O2^{3}$	2.436	Ce1	O2w	2.539
Ce1	$O5^{2}$	2.558			

[1]+X, 1-Y, 1/2+Z, [2]3/2-X, -1/2+Y, 3/2-Z, [3]3/2-X, 1/2-Y, 1-Z.

Supplementary Table 3. Selected bond angles of CeBTTD-B.

Atom1	Atom2	Atom3	Angle/°	Atom1	Atom2	Atom3	Angle/°
$O6^{1}$	Ce1	$O6^{2}$	72.7(4)	$O2^{3}$	Ce1	O3	138.1(4)
$O6^{1}$	Ce1	$O5^{2}$	120.2(4)	$O2^{3}$	Ce1	O1w	71.6(4)
$O6^{1}$	Ce1	O4	133.7(5)	$O2^{3}$	Ce1	O2w	72.3(5)
$O6^{1}$	Ce1	O3	86.9(4)	$O5^{2}$	Ce1	$O6^{2}$	49.3(4)
$O6^{1}$	Ce1	O1w	83.1(4)	$O5^{2}$	Ce1	O4	82.3(5)
$O6^{1}$	Ce1	O2w	146.0(4)	$O5^{2}$	Ce1	O3	129.2(4)
O1	Ce1	$O6^{2}$	70.1(4)	O4	Ce1	$O6^{2}$	126.1(4)
O1	Ce1	$O6^{1}$	71.7(4)	O4	Ce1	O3	51.7(4)
O1	Ce1	$O5^{2}$	75.7(4)	O3	Ce1	$O6^{2}$	142.6(4)
O1	Ce1	O4	76.8(4)	O1w	Ce1	$O6^{2}$	136.1(4)
O1	Ce1	O3	74.0(4)	O1w	Ce1	$O5^{2}$	148.1(4)
O1	Ce1	O1w	135.5(4)	O1w	Ce1	O4	97.1(4)
O1	Ce1	O2w	140.8(4)	O1w	Ce1	O3	68.6(4)
$O2^{3}$	Ce1	$O6^{1}$	76.0(4)	O1w	Ce1	O2w	75.6(4)
$O2^{3}$	Ce1	$O6^{2}$	67.4(3)	O2w	Ce1	$O6^{2}$	105.2(5)
$O2^{3}$	Ce1	O1	132.3(4)	O2w	Ce1	$O5^{2}$	73.3(4)
$O2^{3}$	Ce1	$O5^{2}$	92.0(4)	O2w	Ce1	O4	75.9(6)
$O2^{3}$	Ce1	O4	147.9(4)	O2w	Ce1	O3	108.9(5)

[1]+X, 1-Y, -1/2+Z, [2]3/2-X, -1/2+Y, 3/2-Z, [3]3/2-X, 1/2-Y, 1-Z

Supplementary Table 4. Selected bond lengths (Å) of CeBTTD-N.

Atom1	Atom2	Length/ Å	Atom1	Atom2	Length/ Å
Ce1	O6[2]	2.487	Ce1	O3	2.560
Ce1	O6[3]	2.713	Ce1	O4	2.546
Ce1	O2[1]	2.442	Ce1	O1w	2.509
Ce1	O5[3]	2.521	Ce1	O2w	2.545
Ce1	O1	2.435			

[1]1/2-X, 3/2-Y, 1-Z, [2]+X, 1-Y, -1/2+Z, [3]1/2-X, 1/2+Y, 3/2-Z

Supplementary Table 5. Selected bond angles of CeBTTD-N.

Atom1	Atom2	Atom3	Angle/°	Atom1	Atom2	Atom3	Angle/°
O6[1]	Ce1	O6[2]	72.7(4)	O2[3]	Ce1	O1w	71.6(4)
O6[1]	Ce1	O6[3]	74.3(4)	O1	Ce1	O6[3]	71.1(3)
O6[1]	Ce1	O5[3]	121.7(3)	O1	Ce1	O2[2]	133.1(3)
O6[1]	Ce1	O3	85.2(3)	O1	Ce1	O5[3]	76.4(4)
O6[1]	Ce1	O4	131.7(4)	O1	Ce1	O3	73.4(3)
O6[1]	Ce1	O1w	83.2(4)	O1	Ce1	O4	76.6(4)
O6[1]	Ce1	O2w	146.4(4)	O1	Ce1	O1w	136.3(4)
O2[2]	Ce1	O6[1]	76.6(3)	O1	Ce1	O2w	141.1(4)
O2[2]	Ce1	O6[3]	67.8(3)	O3	Ce1	O6[3]	143.1(3)
O2[2]	Ce1	O5[3]	92.9(3)	O4	Ce1	O6[3]	127.1(4)
O2[2]	Ce1	O3	136.9(3)	O4	Ce1	O3	51.0(4)
O2[2]	Ce1	O4	148.4(4)	O1w	Ce1	O6[3]	135.5(3)
O2[2]	Ce1	O1w	70.0(4)	O1w	Ce1	O5[3]	146.5(4)
O2[2]	Ce1	O2w	72.4(4)	O1w	Ce1	O3	69.3(4)
O5[3]	Ce1	O6[3]	49.5(3)	O1w	Ce1	O4	96.5(4)
O5[3]	Ce1	O3	129.4(3)	O1w	Ce1	O2w	74.4(4)
O5[3]	Ce1	O4	83.2(4)	O2w	Ce1	O6[3]	104.9(4)
O5[3]	Ce1	O2w	73.0(4)	O2w	Ce1	O3	109.0(4)
O1	Ce1	O6[1]	71.5(3)	O2w	Ce1	O4	76.4(5)

[1]+X, 1-Y, -1/2+Z, [2]1/2-X, 3/2-Y, 1-Z, [3]1/2-X, 1/2+Y, 3/2-Z

Supplementary Table 6. Selected bond lengths (Å) of CeBTTD-A.

Atom1	Atom2	Length/ Å	Atom1	Atom2	Length/ Å
Ce1	O9	2.477	Ce1	O7	2.557
Ce1	O10	2.581	Ce1	O4[3]	2.458
Ce1	O3[2]	2.467	Ce1	O8	2.499
Ce1	O6	2.451	Ce1	O11	2.522
Ce1	O6[1]	2.719	Ce1	O12	2.462

[1]1-X, 1-Y, 1-Z, [2]1/2+X, +Y, 1/2-Z, [3]1/2-X, 1-Y, 1/2+Z.

Supplementary Table 7. Selected bond angles of CeBTTD-A.

Atom1	Atom2	Atom3	Angle/°	Atom1	Atom2	Atom3	Angle/°
O9	Ce1	O10	68.5(6)	O7	Ce1	O6[1]	146.2(2)
O9	Ce1	O6[1]	137.6(4)	O4[3]	Ce1	O9	71.1(4)
O9	Ce1	O7	74.8(4)	O4[3]	Ce1	O10	74.2(6)
O9	Ce1	O8	79.3(5)	O4[3]	Ce1	O3[2]	136.2(2)
O10	Ce1	O6[1]	82.0(5)	O4[3]	Ce1	O6[1]	72.0(2)
O10	Ce1	O7	121.9(5)	O4[3]	Ce1	O7	126.2(3)
O3[2]	Ce1	O9	144.5(3)	O4[3]	Ce1	O8	148.8(2)
O3[2]	Ce1	O10	133.3(4)	O4[3]	Ce1	O11	68.7(5)
O3[2]	Ce1	O6[1]	76.2(2)	O4[3]	Ce1	O12	78.2(6)
O3[2]	Ce1	O7	72.2(2)	O8	Ce1	O10	86.3(6)
O3[2]	Ce1	O8	75.0(2)	O8	Ce1	O6[1]	129.8(2)
O3[2]	Ce1	O11	134.6(6)	O8	Ce1	O7	50.9(2)
O6	Ce1	O9	109.8(5)	O8	Ce1	O11	87.3(6)
O6	Ce1	O10	144.9(6)	O11	Ce1	O6[1]	140.7(5)
O6	Ce1	O3[2]	71.9(2)	O11	Ce1	O7	64.4(5)
O6	Ce1	O6[1]	77.6(2)	O12	Ce1	O3[2]	139.0(6)
O6	Ce1	O7	81.8(2)	O12	Ce1	O6[1]	103.3(7)
O6	Ce1	O4[3]	72.5(3)	O12	Ce1	O7	108.2(7)
O6	Ce1	O8	128.6(2)	O12	Ce1	O8	75.1(7)
O6	Ce1	O11	89.0(6)	O12	Ce1	O11	70.4(8)
O6	Ce1	O12	148.8(6)				

[1]1-X,1-Y,1-Z, [2]1/2+X,+Y,1/2-Z, [3]1/2-X,1-Y,1/2+Z

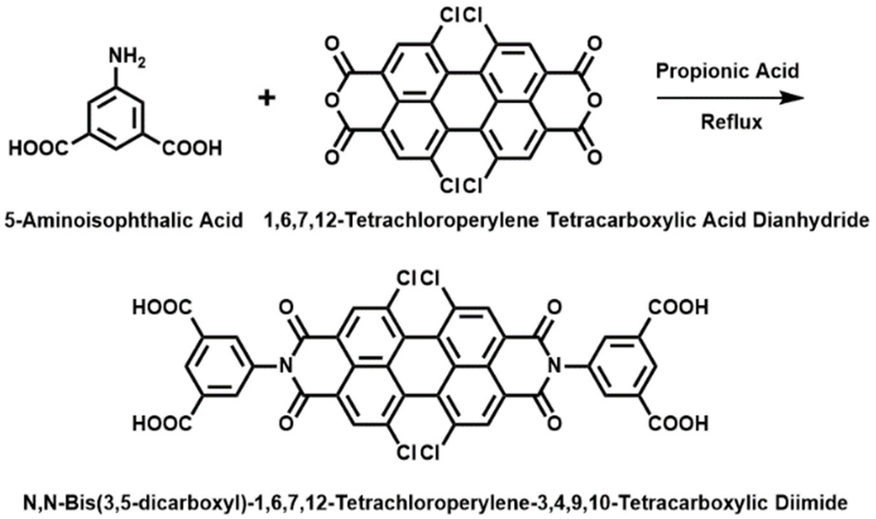

Supplementary Figure 1. The synthetic route of H_4BTTD.

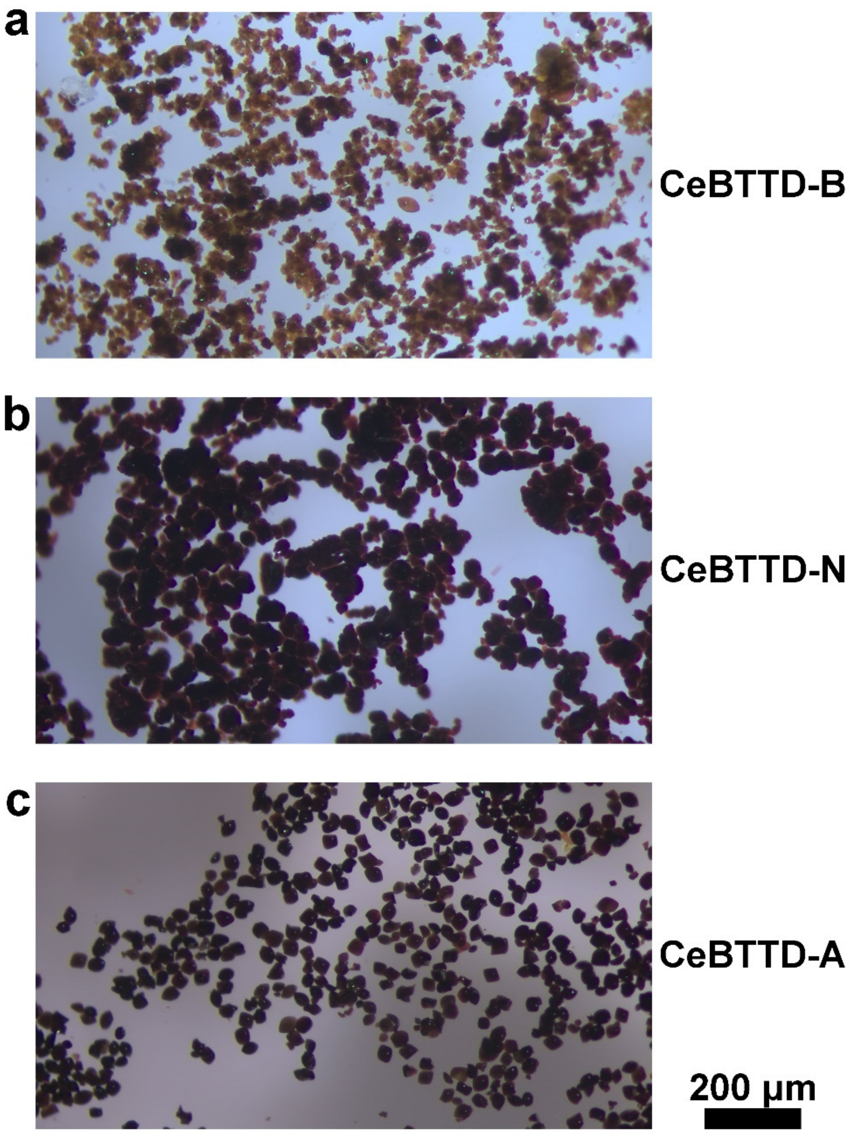

Supplementary Figure 2. The photograph of **a** CeBTTD-B, **b** CeBTTD-N and **c** CeBTTD-A under an optical microscope.

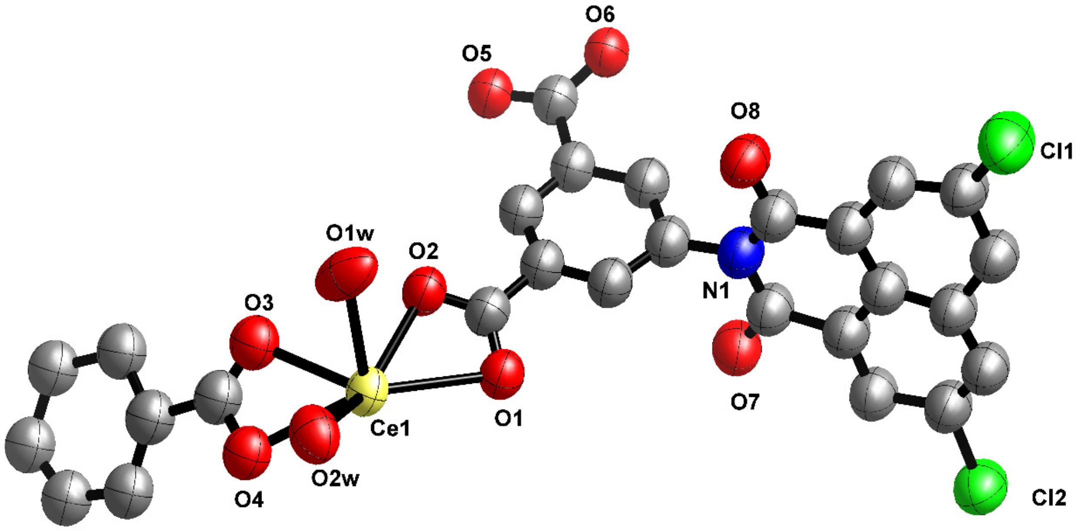

Supplementary Figure 3. Asymmetric unit in the single-crystal structure of CeBTTD-B. Thermal ellipsoids are drawn with 50% probability. All hydrogen atoms are omitted for clarity. Ce, yellow, Cl, green, O, red, C, gray, N, blue.

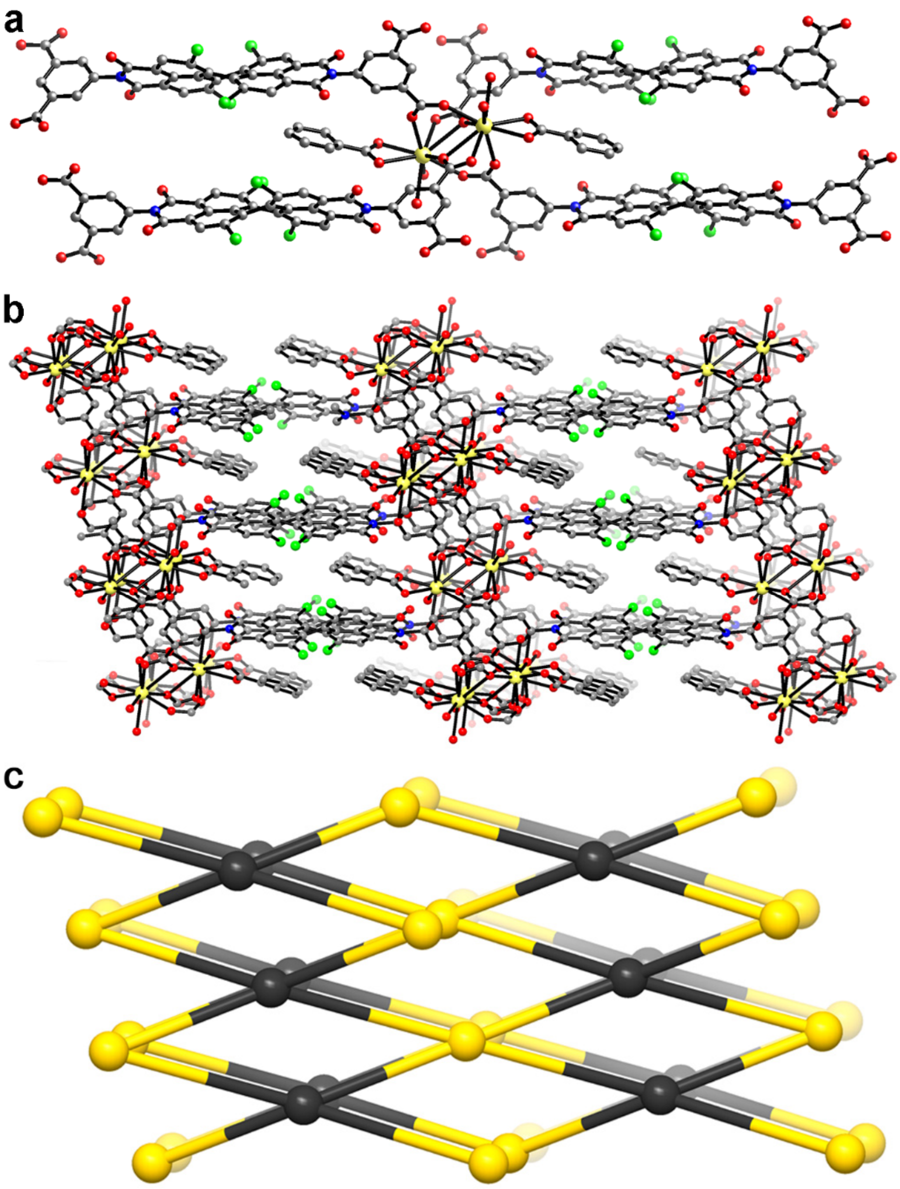

Supplementary Figure 4. a The coordination environment of Ce_2 in CeBTTD-B. Each Ce_2 in CeBTTD-B was coordination with four $BTTD^{4-}$, two BA^- and two coordinated water molecules. All hydrogen atoms are omitted for clarity. Ce, yellow, Cl, green, O, red, C, gray, N, blue. **b** The three-dimensional structural and **c** the corresponding topology diagrams of CeBTTD-B, which the yellow balls represent $\{Ce_2\}$ and black balls represent $BTTD^{4-}$, respectively.

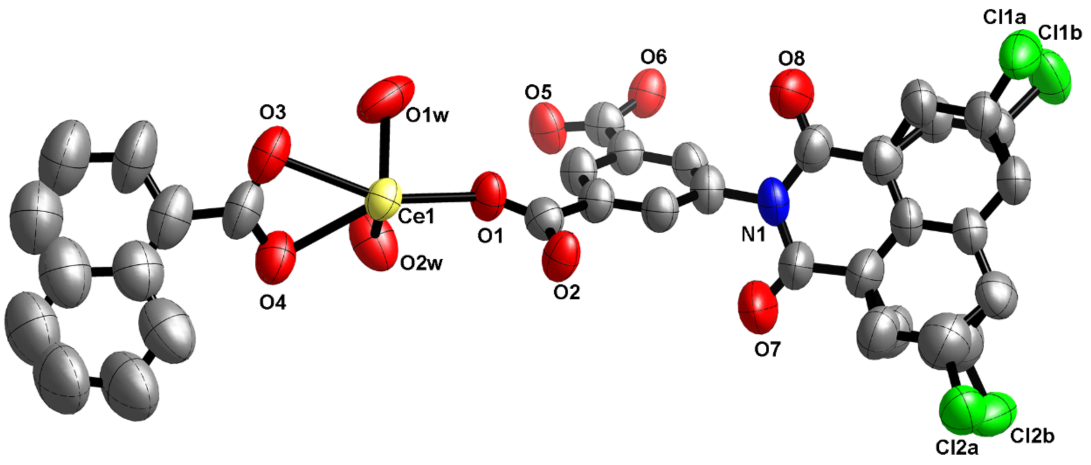

Supplementary Figure 5. Asymmetric unit in the single-crystal structure of CeBTTD-N with the disordered ligands. Thermal ellipsoids are drawn with 50% probability. All hydrogen atoms are omitted for clarity. Ce, yellow, Cl, green, O, red, C, gray, N, blue.

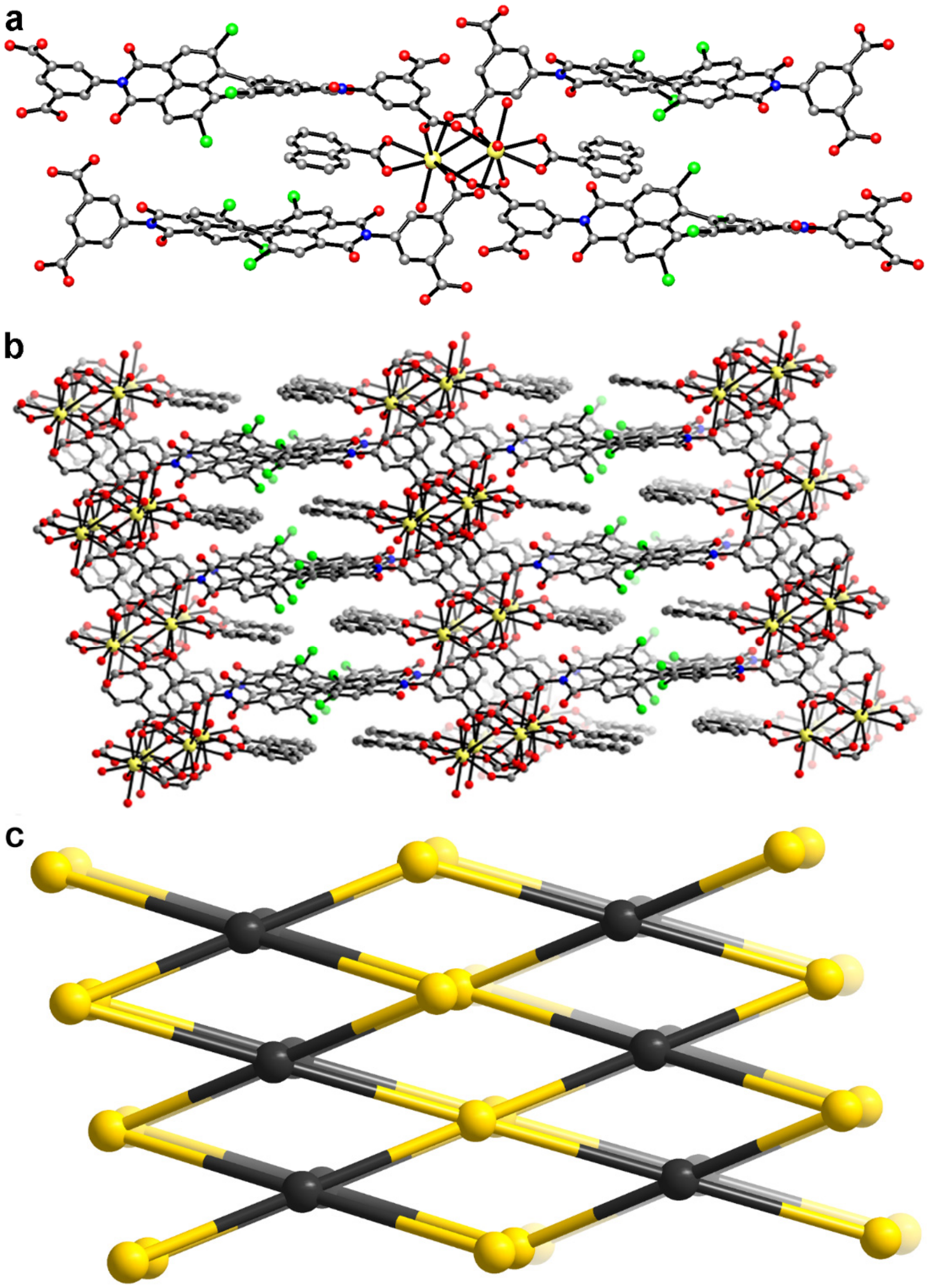

Supplementary Figure 6. a The coordination environment of Ce_2 in CeBTTD-N. Each Ce_2 in CeBTTD-N was coordination with four $BTTD^{4-}$, two NA^- and two coordinated water molecules. All hydrogen atoms are omitted for clarity. Ce, yellow, Cl, green, O, red, C, gray, N, blue. **b** The three-dimensional structural and **c** the corresponding topology diagrams of CeBTTD-N, which the yellow balls represent $\{Ce_2\}$ and black balls represent $BTTD^{4-}$, respectively.

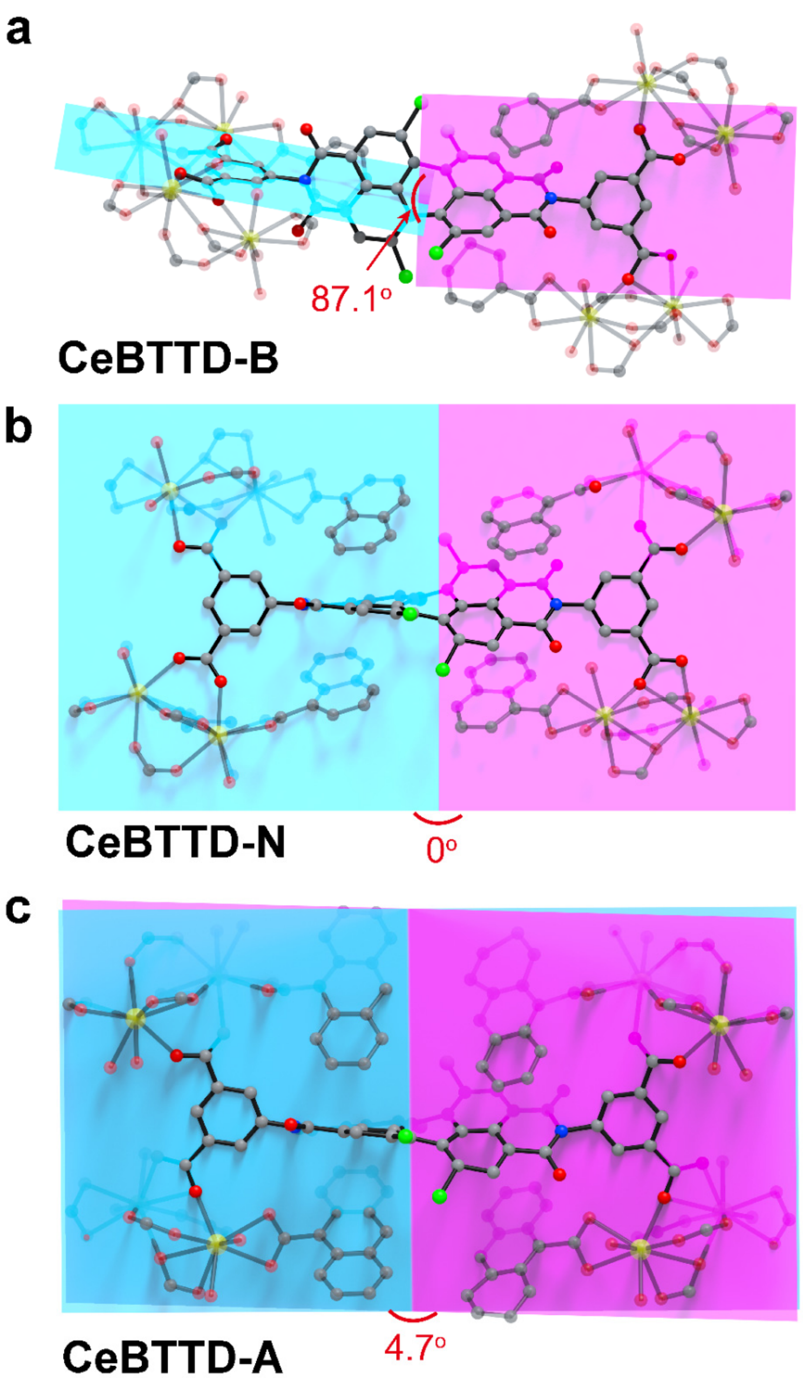

Supplementary Figure 7. The dihedral angle between two benzene rings in $BTTD^{4-}$ ligand in CeBTTD HMMJs. **a** The planes of the two benzene rings in $BTTD^{4-}$of CeBTTD-B are almost perpendicular, and the dihedral angle is calculated to be 87.1 degree. **b** The planes of the two benzene rings in $BTTD^{4-}$ of CeBTTD-N are completely coincident, and the dihedral angle is 0 degree. **c** There is only a very small angle between the two planes of the benzene rings in $BTTD^{4-}$ of CeBTTD-A and the dihedral angle is calculated to be 4.7 degrees. All hydrogen atoms are omitted for clarity.

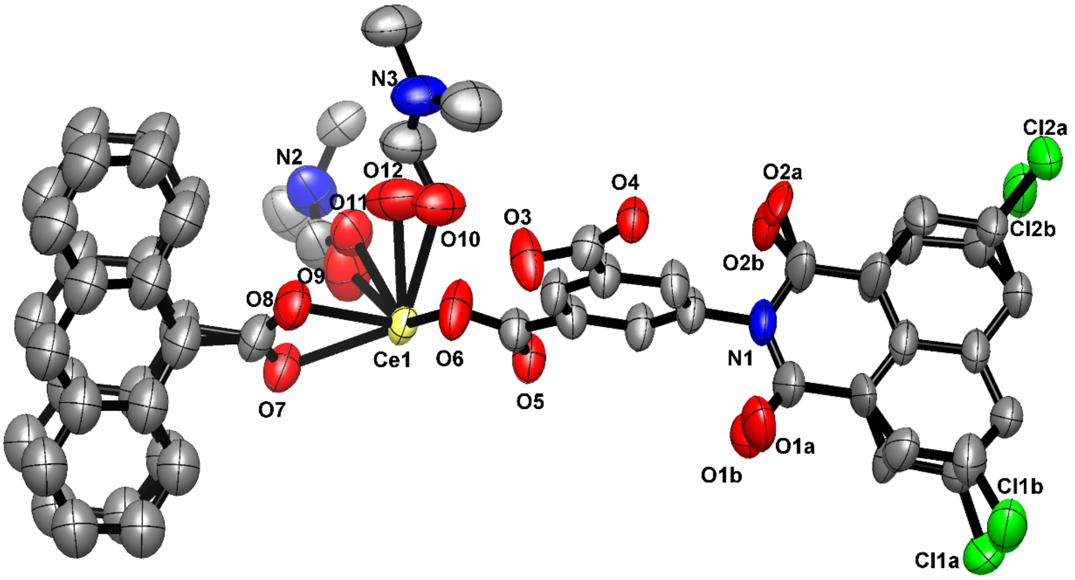

Supplementary Figure 8. Asymmetric unit in the single-crystal structure of CeBTTD-A with the disordered ligands and solvent molecules. Thermal ellipsoids are drawn with 50% probability. All hydrogen atoms are omitted for clarity. Ce, yellow, Cl, green, O, red, C, gray, N, blue.

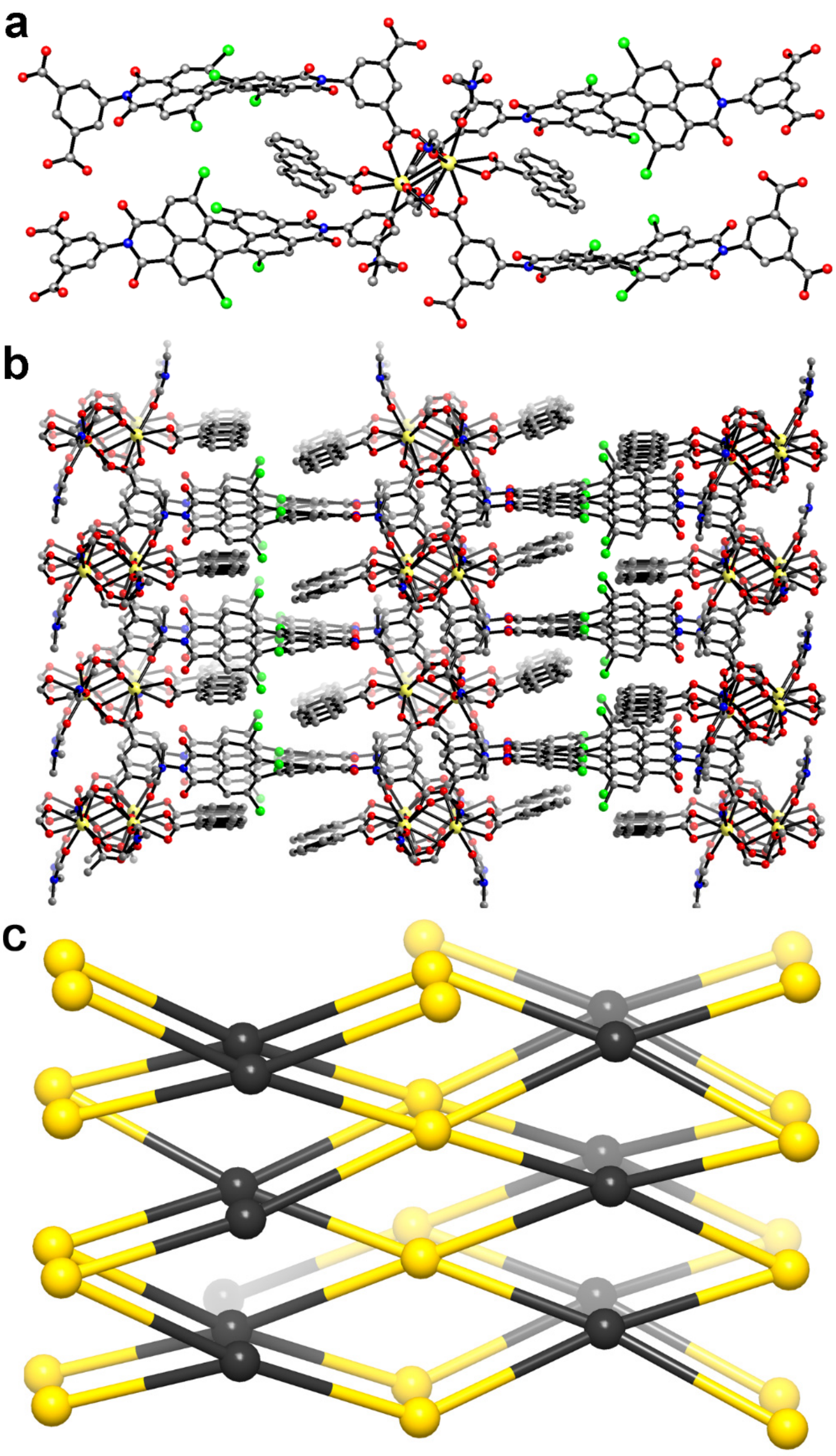

Supplementary Figure 9. a The coordination environment of Ce_2 in CeBTTD-A. All hydrogen atoms are omitted for clarity. Ce, yellow, Cl, green, O, red, C, gray, N, blue. **b** The three-dimensional structural and **c** the corresponding topology diagrams of CeBTTD-A, which the yellow balls represent $\{Ce_2\}$ and black balls represent $BTTD^{4-}$, respectively.

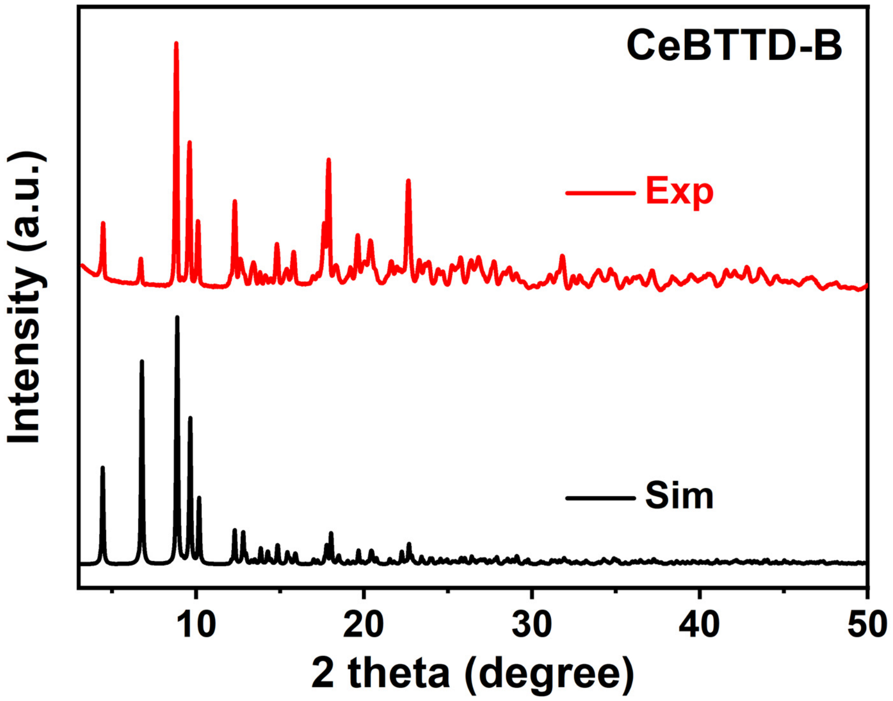

Supplementary Figure 10. The experimental (red) and simulated (black) PXRD patterns of CeBTTD-B.

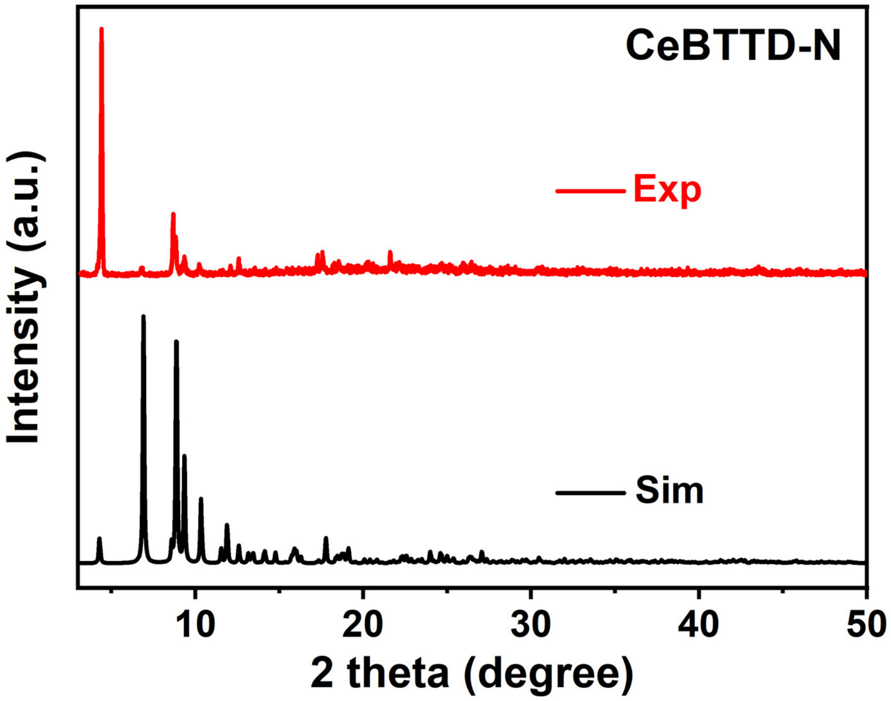

Supplementary Figure 11. The experimental (red) and simulated (black) PXRD patterns of CeBTTD-N.

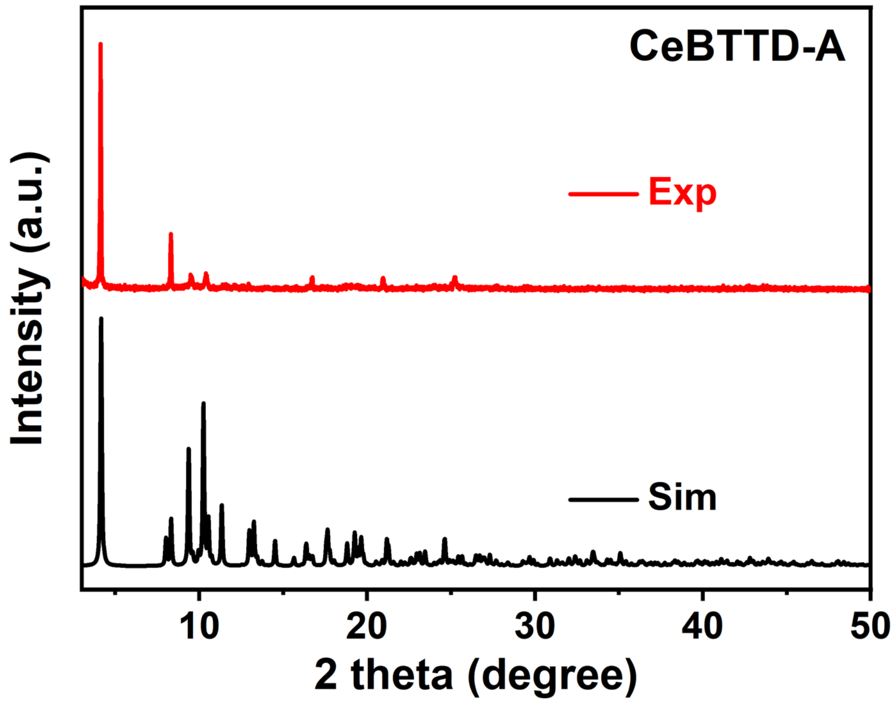

Supplementary Figure 12. The experimental (red) and simulated (black) PXRD patterns of CeBTTD-A.

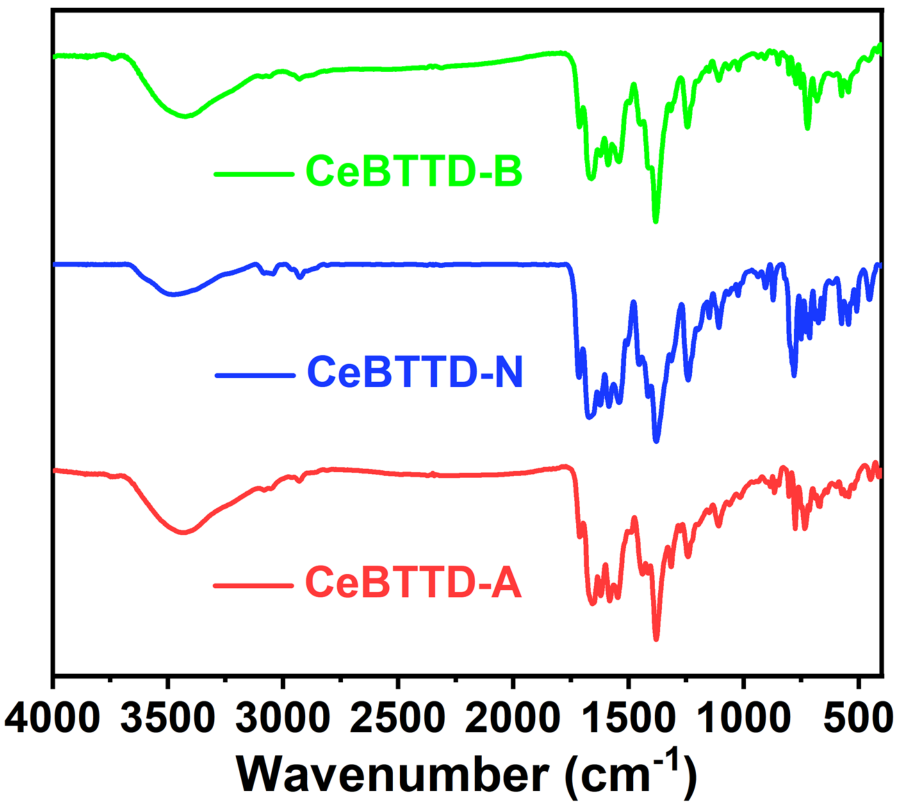

Supplementary Figure 13. The FTIR spectra of CeBTTD-B (green), CeBTTD-N (blue) and CeBTTD-A (red), respectively.

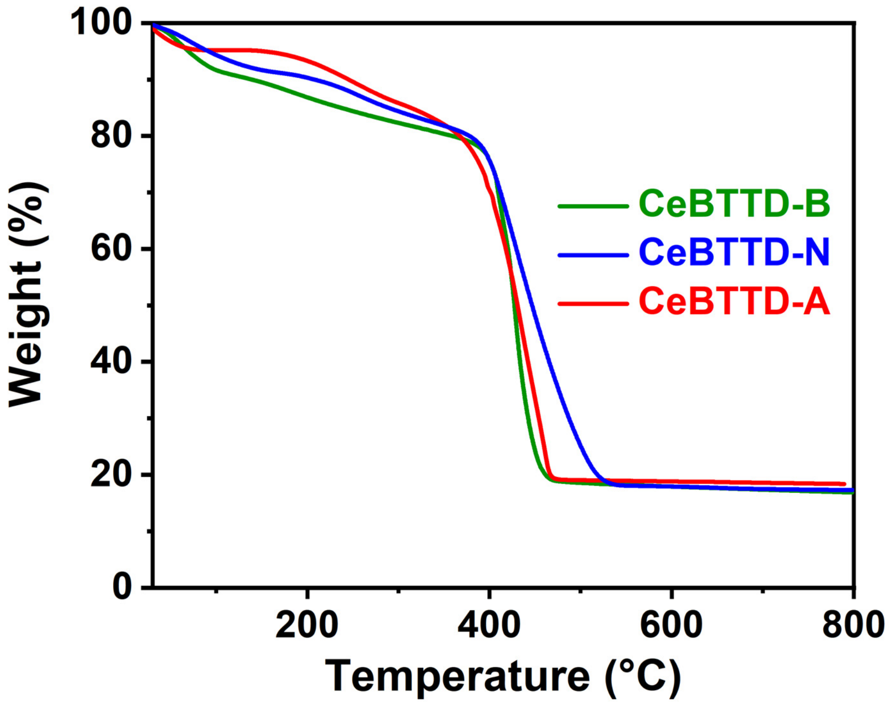

Supplementary Figure 14. The TGA curves of CeBTTD-B (green), CeBTTD-N (blue) and CeBTTD-A (red), respectively.

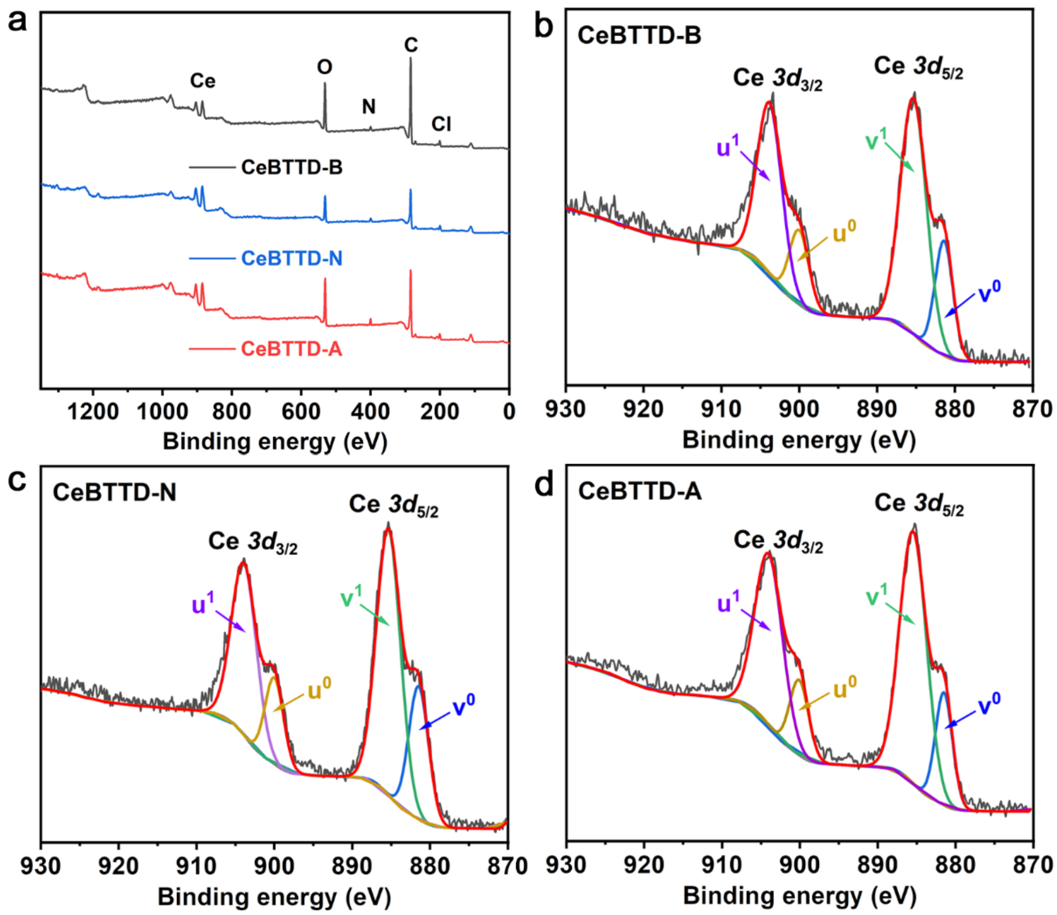

Supplementary Figure 15. a XPS survey spectra and High-resolution peaks of Ce *3d* spectrum of **b** CeBTTD-B, **c** CeBTTD-N and **d** CeBTTD-A, respectively. There is no satellite peak located around 916.0 eV, which suggest that Ce ions in CeBTTD HMMJs are all in +3 oxidation state.[1]

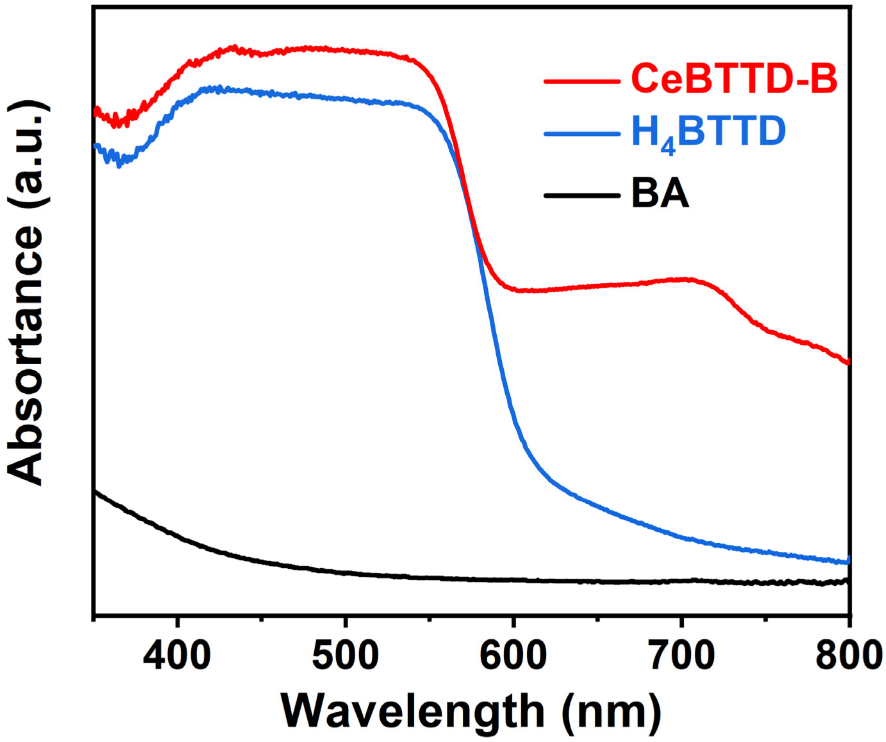

Supplementary Figure 16. UV-Visible diffuse reflection spectra for CeBTTD-B and its motifs (BA and H_4BTTD).

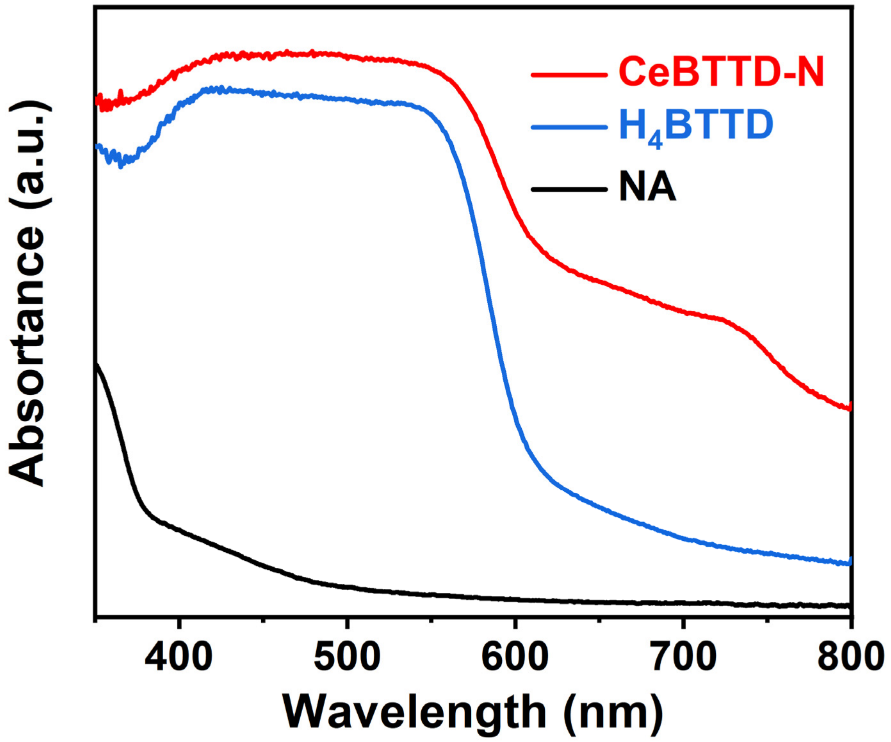

Supplementary Figure 17. UV-Visible diffuse reflection spectra for CeBTTD-N and its motifs (NA and H_4BTTD).

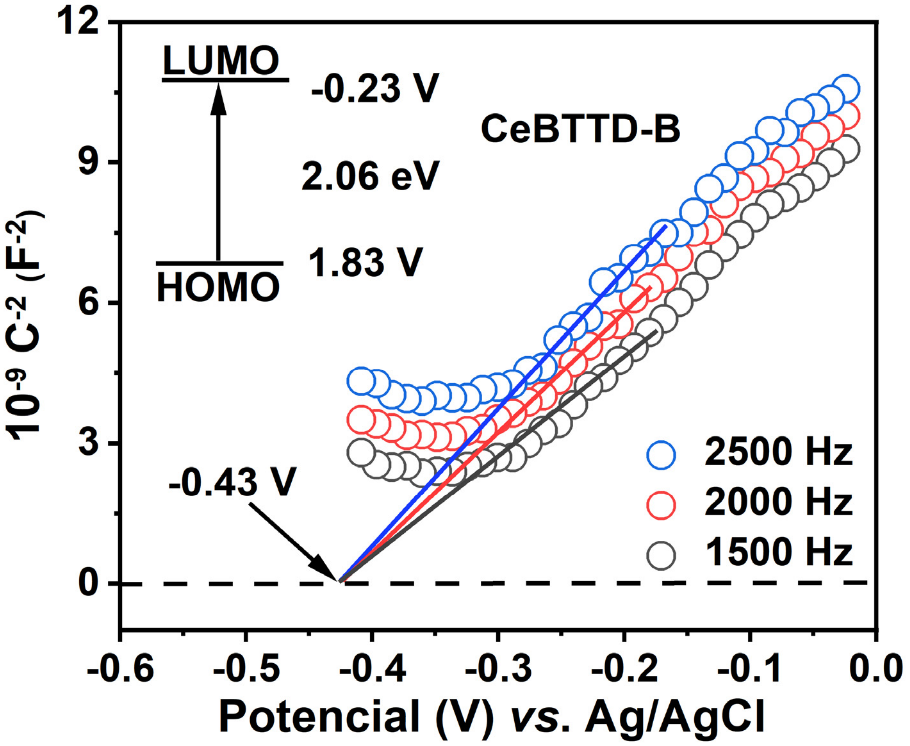

Supplementary Figure 18. Mott-Schottky plot measurement for CeBTTD-B. Inset: Energy diagram of the HOMO and LUMO levels of CeBTTD-B.

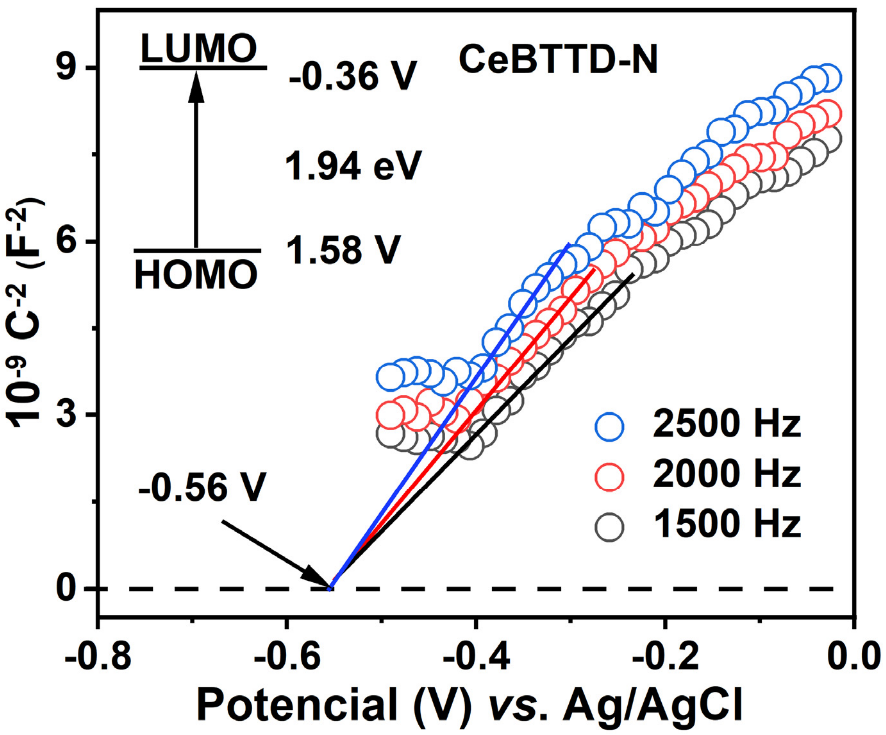

Supplementary Figure 19. Mott-Schottky plot measurement for CeBTTD-N. Inset: Energy diagram of the HOMO and LUMO levels of CeBTTD-N.

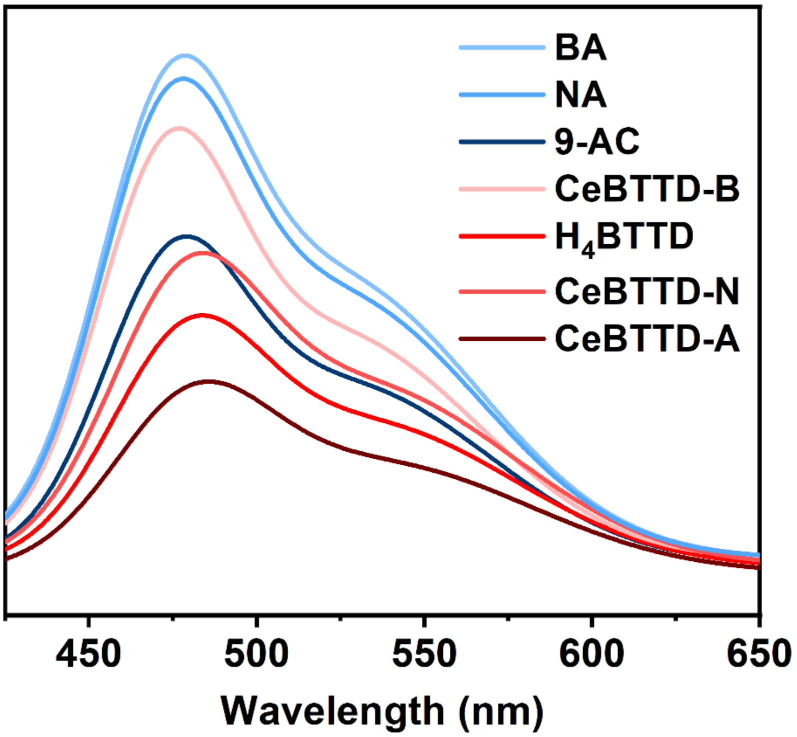

Supplementary Figure 20. Photoluminescence (PL) emission spectra of CeBTTD HMMJs and ligands under excitation at 400 nm.

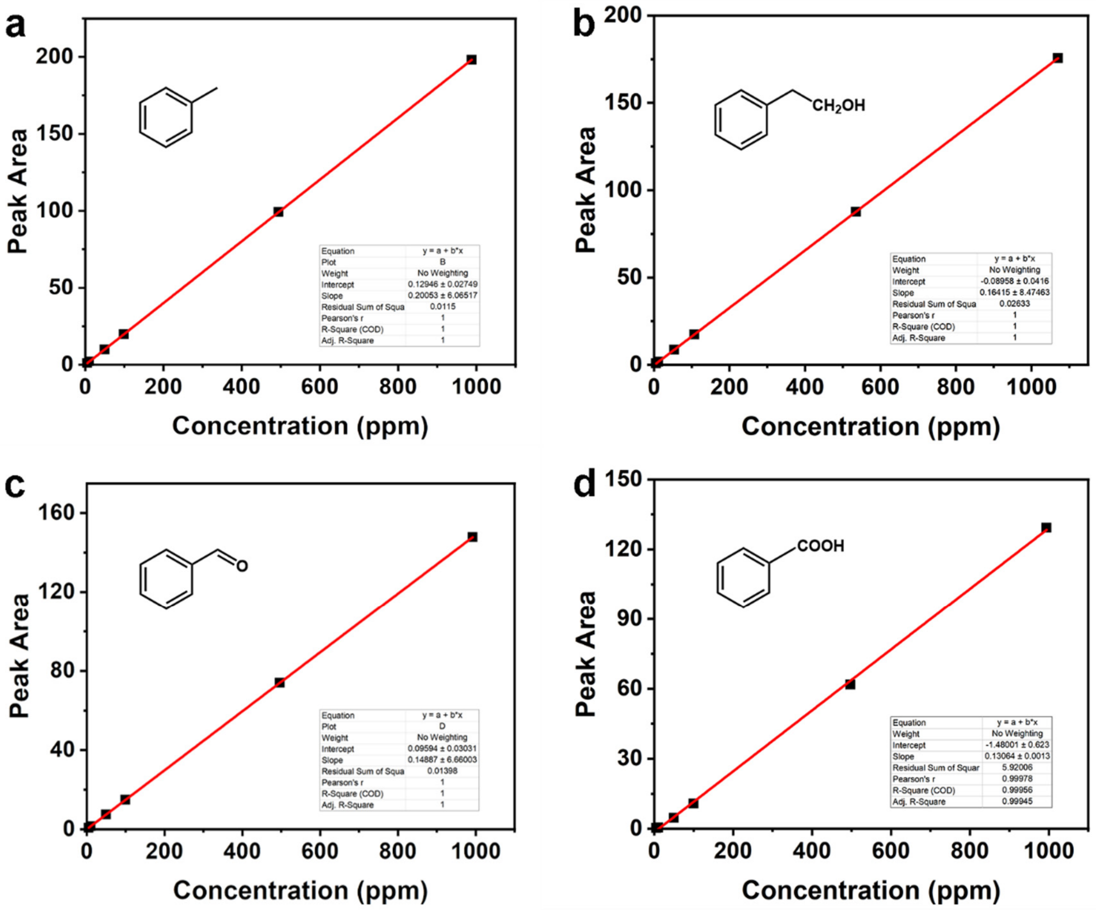

Supplementary Figure 21. Standard curves of **a** toluene, **b** benzyl alcohol, **c** benzaldehyde and **d** benzoic acid by GC analysis.

Supplementary Table 8. The photocatalytic performance of toluene oxidation with different photocatalysts.

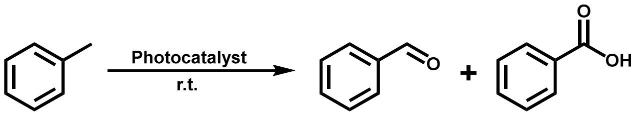

Entry	Photocatalyst	Reaction Condition	Conversion (%)
1	CeBTTD-A	normal	99
2	CeBTTD-N	normal	trace
3	CeBTTD-B	normal	trace
4	CeBTTD-A	no substrate	trace
5	CeBTTD-A	N_2 replace O_2	trace
6	no catalyst	normal	trace
7	CeBTTD-A	dark	trace
8	9-AC (homogeneous photocatalyst)	normal	48
9	H_4BTTD	normal	trace
10	H_4BTTD : 9-AC (2 mg : 1 mg)	normal	70
11	H_4BTTD : 9-AC : $CeCl_3$ (2 mg : 1 mg : 2 mg)	normal	72

Normal condition: 5 mg photocatalyst, 0.1 mmol substrate, 24 hours, 3 mL CH_3CN as the reagent, 300 W xenon light irradiation (300-1100 nm), 1 atm O_2.

Supplementary Table 9. Photocatalytic performance of oxidation of toluene derivatives with CeBTTD-A as the photocatalyst.

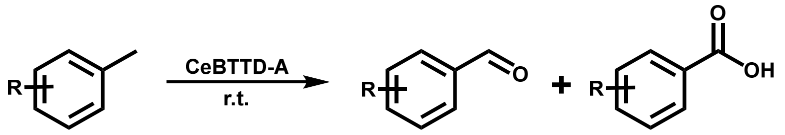

R	Atmosphere	Yield (%) R-ph-CHO	Yield (%) R-ph-COOH	Conversion (%)
p-H	O_2	trace	98	98
	Air	2	93	95
p-OCH_3	O_2	trace	99	99
	Air	trace	97	97
p-CN	O_2	10	62	72
	Air	7	50	57
p-F	O_2	trace	98	98
	Air	2	96	98
p-Cl	O_2	3	95	98
	Air	19	66	85
p-Br	O_2	13	76	89
	Air	22	53	75
o-Br	O_2	22	55	77
	Air	10	45	55
				**COOH-ph-COOH**
p-CH_3	O_2	trace	trace	74
	Air	trace	trace	53

Condition: 5 mg photocatalyst, 0.1 mmol substrate, 24 hours, 3 mL CH_3CN as the reagent, 300 W xenon light irradiation (300-1100 nm).

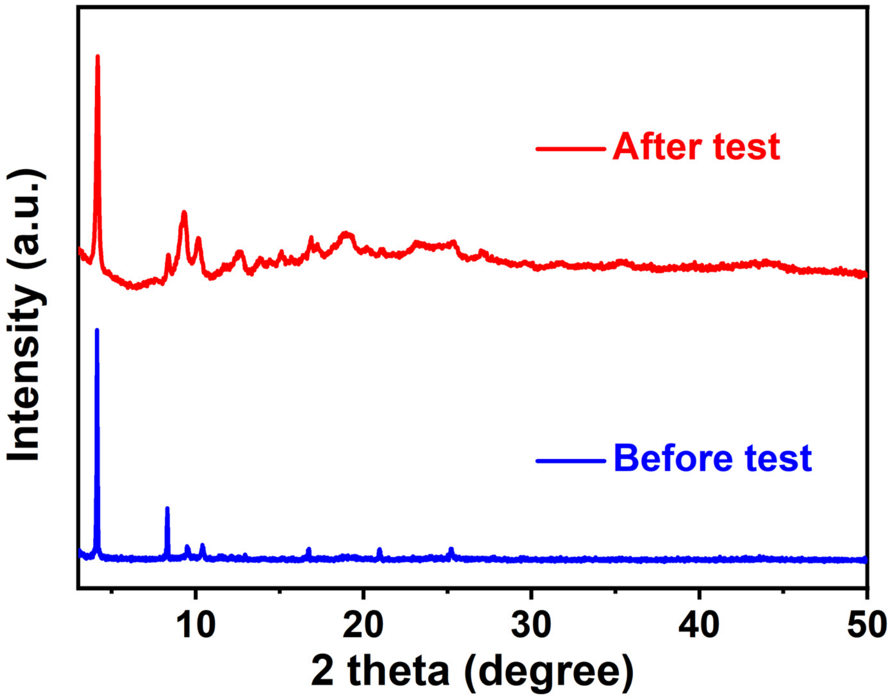

Supplementary Figure 22. The PXRD pattern of CeBTTD-A before (blue) and after (red) the photocatalytic tests.

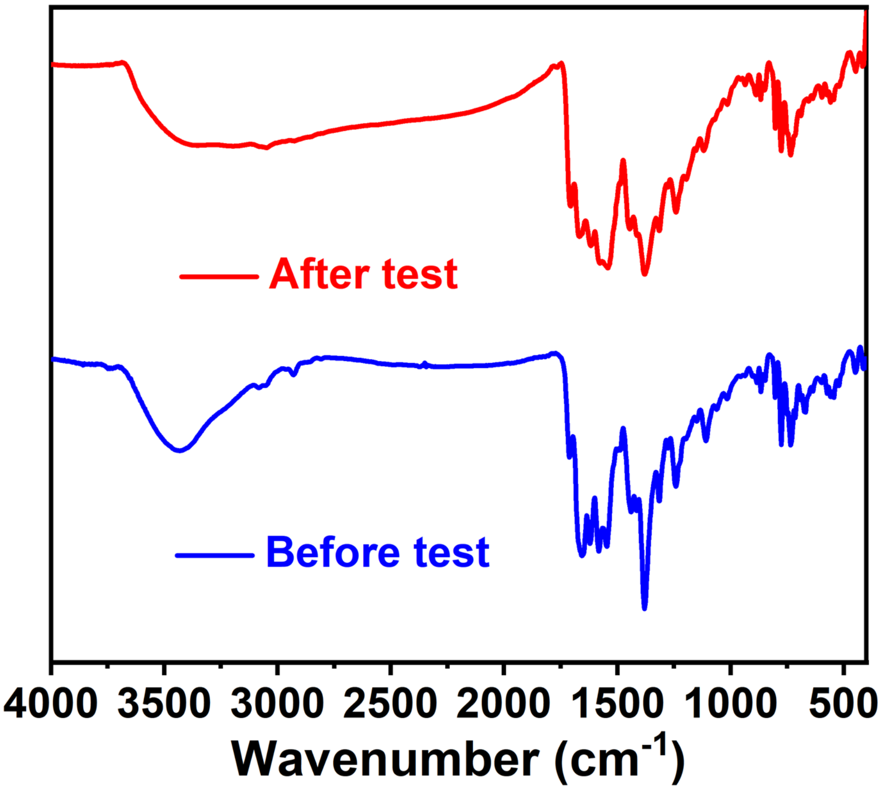

Supplementary Figure 23. The FTIR spectra for CeBTTD-A before (blue) and after (red) the photocatalytic tests.

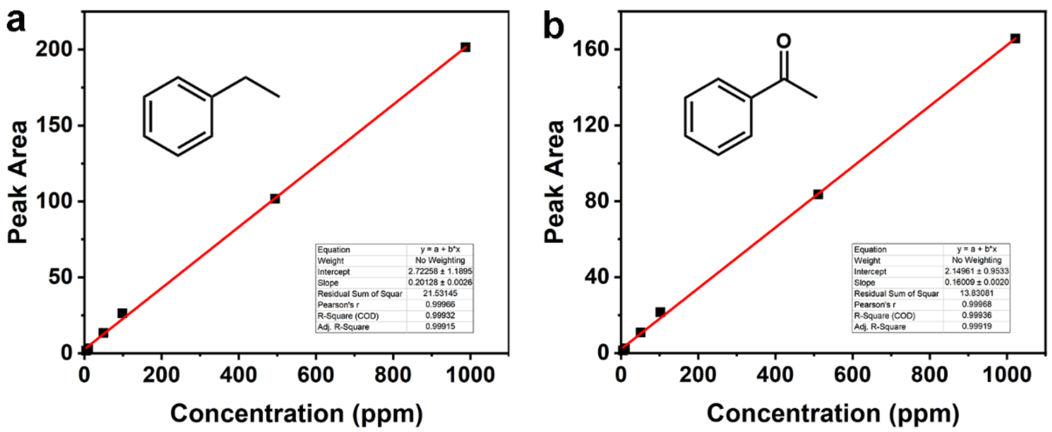

Supplementary Figure 24. Standard curves of **a** ethylbenzene and **b** acetophenone by GC analysis.

Supplementary Table 10. The photocatalytic performance of ethylbenzene oxidation with different photocatalysts.

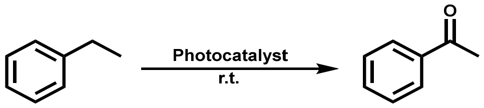

Entry	Photocatalyst	Reaction Condition	Conversion (%)
1	CeBTTD-A	normal	99
2	CeBTTD-N	normal	1
3	CeBTTD-B	normal	trace
4	CeBTTD-A	no substrate	trace
5	CeBTTD-A	N_2 replace O_2	trace
6	no catalyst	normal	trace
7	CeBTTD-A	dark	trace
8	9-AC (homogeneous photocatalyst)	normal	35
9	H_4BTTD	normal	trace
10	H_4BTTD : 9-AC (2 mg : 1 mg)	normal	65
11	H_4BTTD : 9-AC : $CeCl_3$ (2 mg : 1 mg : 2 mg)	normal	63

Normal condition: 5 mg photocatalyst, 0.1 mmol substrate, 12 hours, 3 mL CH_3CN as the reagent, 300 W xenon light irradiation (300-1100 nm), 1 atm O_2.

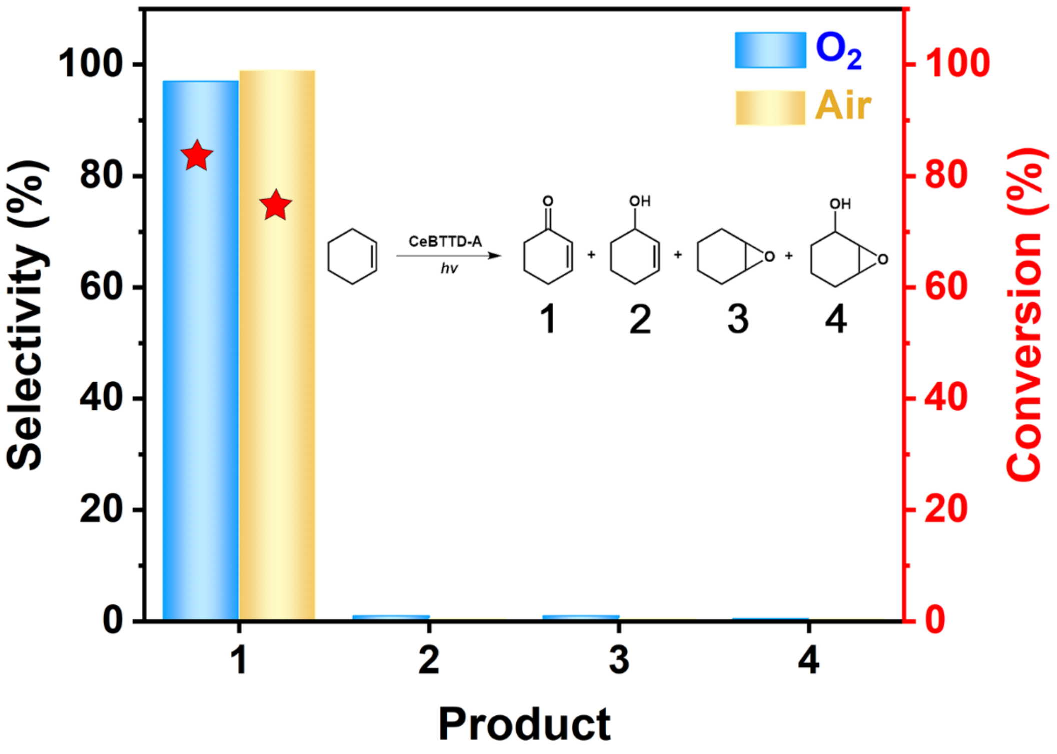

Supplementary Figure 25. Photocatalytic performance for aerobic oxidation of cyclohexene. Reaction condition: 0.1 mmol substrates, 5 mg CeBTTD-A as the photocatalyst, air or O_2 as reaction atmosphere, 24 hours, 3 mL CH_3CN as the reagent, 300-1100 nm xenon light source.

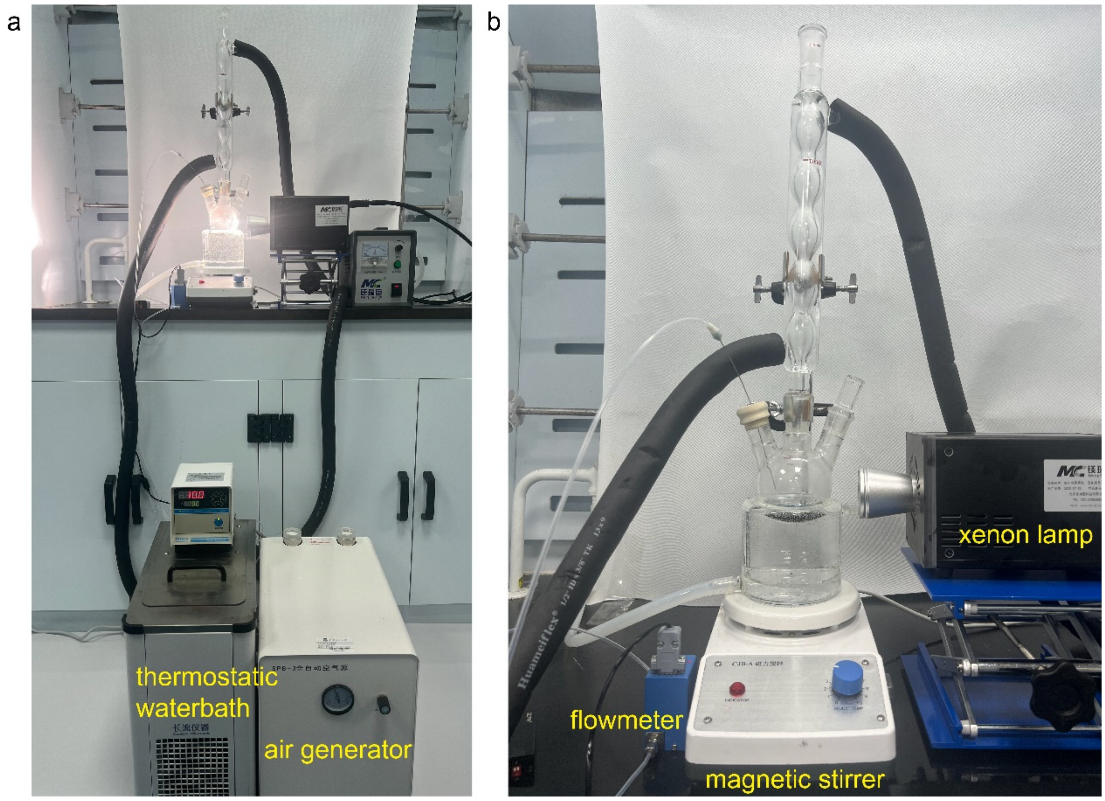

Supplementary Figure 26. a The reaction equipment and **b** the illumination equipment for the solvent-free ten-gram-scale reaction of ethylbenzene oxidation. The photocatalytic experiment was carried out on a 300 W xenon lamp (λ=300~1100 nm). Before the catalytic tests, 100 mg CeBTTD-A as the photocatalyst and 10 g ethylbenzene were added in 250 mL three-necked flask. Through controlling by air generator and flowmeter, fresh air is injected into the reaction system at a flow rate of 1 mL/min. Meanwhile, the reaction environment was controlled at room temperature by using thermostatic waterbath and cooling water circulation.

Supplementary Table 11. Comparison of the photocatalytic aerobic oxidation of ethylbenzene activities of the reported heterogeneous materials.

	Catalyst	Oxidant	Tempe-rature (ºC)	Pressure (bar)	Solvent	Ethyl-benzene (mmol)	Yield		Illumination	Ref.
							%	μmol/g		
1	CeBTTD-A	Air	rt.	1	Solvent-free	94.2	91.4	861	300-780 nm	This work
2	CF_3SO_2Na	O_2	rt.	1	CH_3CN	10	89	22.81	400-405 nm blue light	2
3	Chlorinated $BiOBr/TiO_2$	O_2	rt.	1	Solvent-free	8.17		14.21	420-780 nm	3
4	VO@g-C_3N_4	H_2O_2	rt.	1	CH_3CN	1	99	39.62	40 W domestic bulb	4
5	[Fe(TPA)(MeCN)$_2$](ClO_4)$_2$/RFT	Air	rt.	1	CH_3CN, H_2O, $HClO_4$	0.02	74	62.8	440 nm	5
6	$Cs_3Bi_2Br_9$/SBA-15	Air	rt.	1	Solvent-free	41.03	0.16	65.8	≥420 nm	6
7	TTMBPY·3Br	Air	rt.	1	CH_3CN	0.1	82	64.06	365 nm LED	7
8	[BSPy][Otf]	O_2	rt.	1	CH_3CN	1	92	50.36	365 nm	8
9	*p*-BiOBr	O_2	rt.	1	H_2O, t-BuOH	0.2	93	9.3	>400 nm	9
10	*a*-Fe_2O_3	O_2	40	1	CH_3CN, NHPI	0.1	74	7.4	455 nm LED	10
11	Cercosporin	O_2	rt.	1	KBr, CH_3OH	0.25	78	73.03	23 W CFL	11
12	Imide-acridinium salt PC III	Air	rt.	1	CH_3CN, H_2O	0.1	96	24.62	457 nm LEDs	12
13	SA-Fe-TCN	O_2	60	1	CH_3CN, NHPI, TEA	0.5	99	9.9	> 420 nm	13
14	Cu-PMOF-3 (Fe)	Air	60	1	CH_3CN, NHPI	0.2	82	41		14
15	bisCu	O_2	70	5	CH_3CN, NHPI	5	72	72		15
16	Mo(VI)-V(V)	O_2	75	1	CH_3COOH, NHPI	1	80	61.54		16
17	Pt-BNP	O_2, TBHP	80	1	H_2O	9.43	93	58.13		17

18	Bioderived $Cu_3(PO_4)_2$	O_2	100	40	CH_3CN, NHPI	1	93	9.3		18
19	$(La,Sr)_{0.5}$-$(Mn,Co)_{0.5}O_{2.38}$	O_2	90	20	CH_3COOH, NHPI	13.75	37	101.75		19
20	LC-N-8.9	TBHP	80	1	H_2O	1	91	91.3		20
21	MFM-170	TBHP	65	1	CH_3CN	0.25	30	3		21
22	rac-1	H_2O_2	rt.	1	CH_3COOH, CH_3CN	0.5	82	53.95		22
23	SDC-A	TBHP	80	1	Solvent-free	1	93	46.5		23
24	DACAQ/NHPI/HY	O_2	80	3	CH_3CN, NHPI	16.41	61	79.48		24
25	Fe-N-C-700	TBHP	rt.	1	H_2O	0.5	99	49.5		25
26	$[Fe(qpy)](ClO_4)_2$	Oxone	80	1	CH_3CN, H_2O	0.2	41	11.33		26
27	0.5%Pd@C-GluA-550	Air	120	1	Solvent-free	32.83	13	219.14		27
28	$[(pymox-Me_2)RuCl_2]^+BF^{4-}$	t-BuOOH	rt.	1	H_2O	1	83	136.07		28

The green area represents the photocatalytic systems, the blue area represents the thermocatalytic systems.

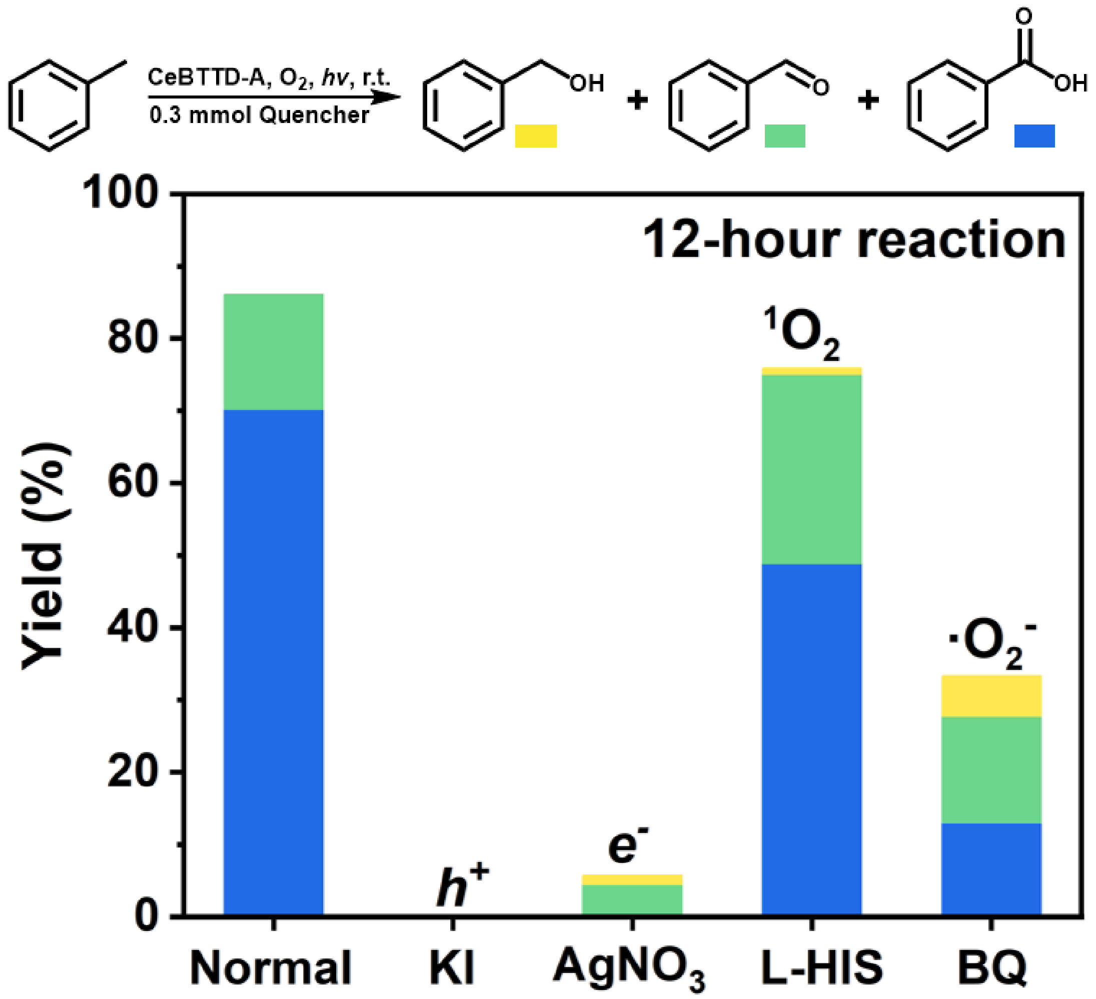

Supplementary Figure 27. Control experiments for photocatalytic toluene oxidation by CeBTTD-A under normal condition or with different quenchers.

Normal condition: 5 mg photocatalyst, 0.1 mmol substrate, 4 hours, 3 mL CH_3CN as the reagent, 300 W xenon light irradiation (300-1100 nm), 1 atm O_2.

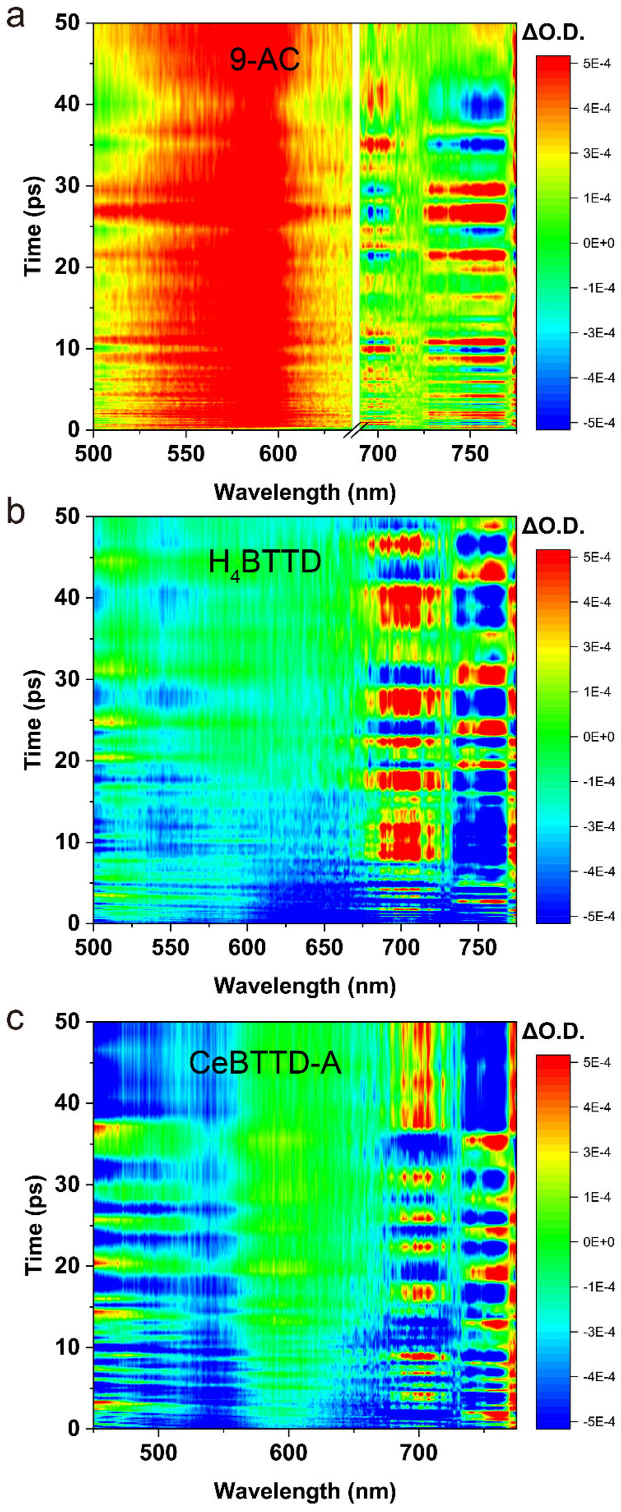

Supplementary Figure 28. TA spectra of 9-AC, H_4BTTD and CeBTTD-A probed within the region of 450-780 nm. excitation wavelength: **a** 9-AC (340 nm), **b** H_4BTTD (400 nm) and **c** CeBTTD-A (400 nm).

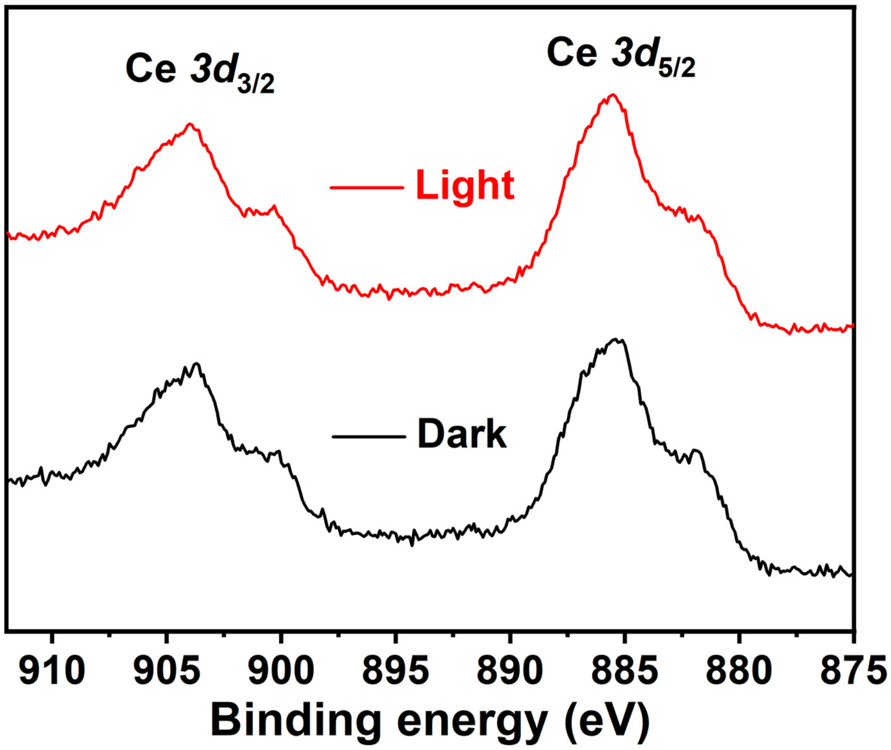

Supplementary Figure 29. In situ high-resolution Ce *3d* XPS spectra of CeBTTD-A in dark (black curve) and light (red curve) conditions.

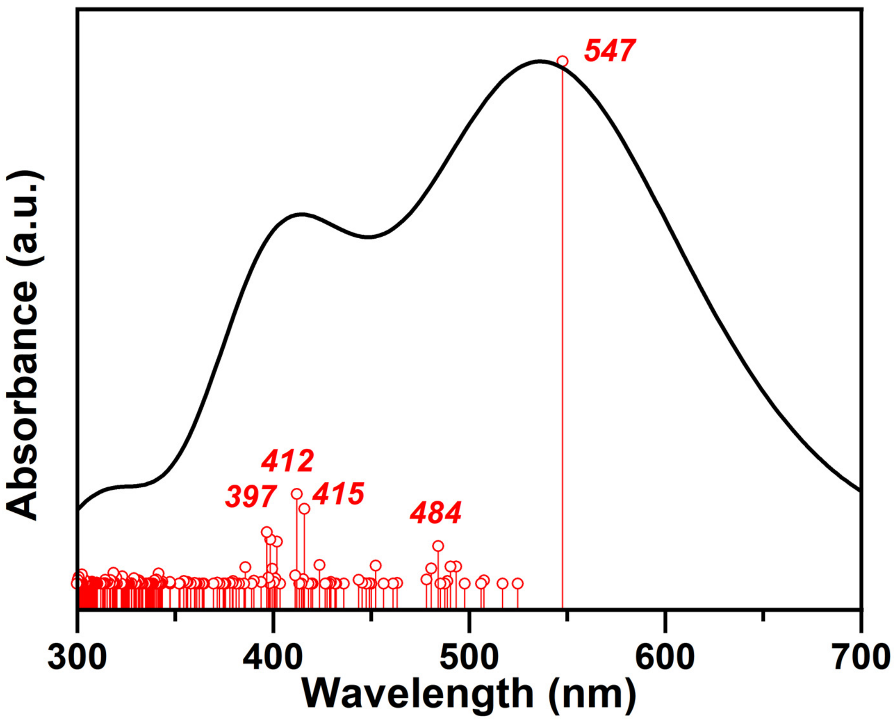

Supplementary Figure 30. Calculated absorption spectrum of CeBTTD-A.

Supplementary Table 12. Absorption peaks λ_{abs} [in eV (nm)], the oscillator strengths (*f*), major contributions and assignments (H and L represent HOMO and LUMO, respectively).

λ_{abs}	***f***	**Major Contributions**	**Major Assignments**
547	0.428	H-3→L (96%),	BTTD→BTTD
484	0.031	H-1→L+1 (49%)	9-AC→BTTD
415	0.062	H→L+2 (76%)	9-AC→9-AC
412	0.074	H-1→L+2 (78%)	9-AC→9-AC
397	0.042	H-1→L+4 (86%)	9-AC→9-AC

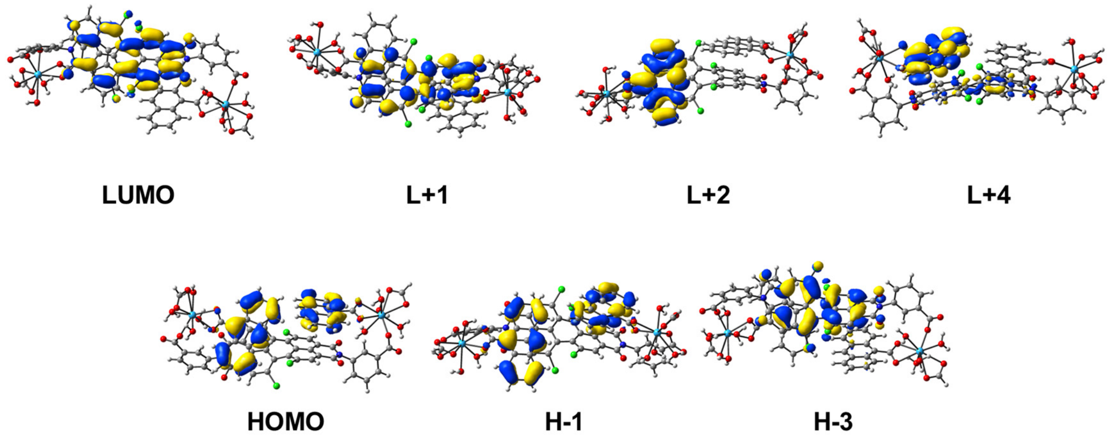

Supplementary Figure 31. The calculated major frontier molecular orbital distributions involved in excitation process of Supplementary Table 12**.**

^1H NMR and GC-MS analysis of products.

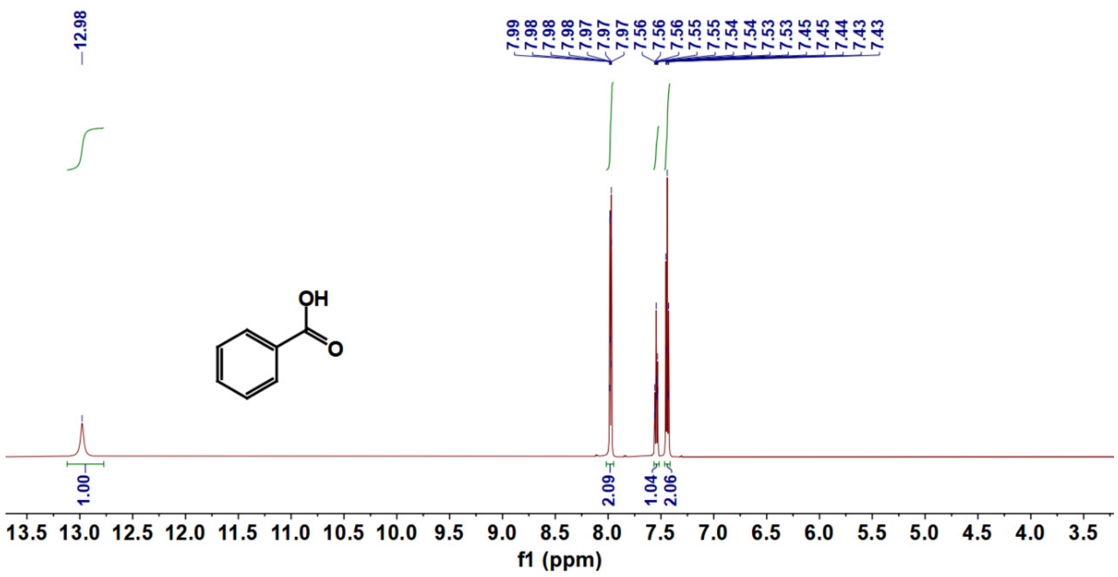

Supplementary Figure 32. ^1H NMR spectrum of the reaction product using toluene as substrate in Figure 4a.

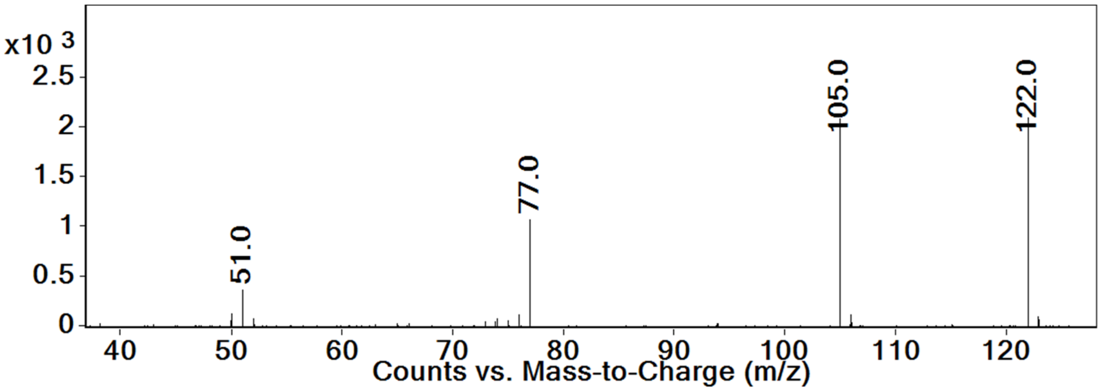

Supplementary Figure 33. Mass spectrum of the reaction product using toluene as substrate.

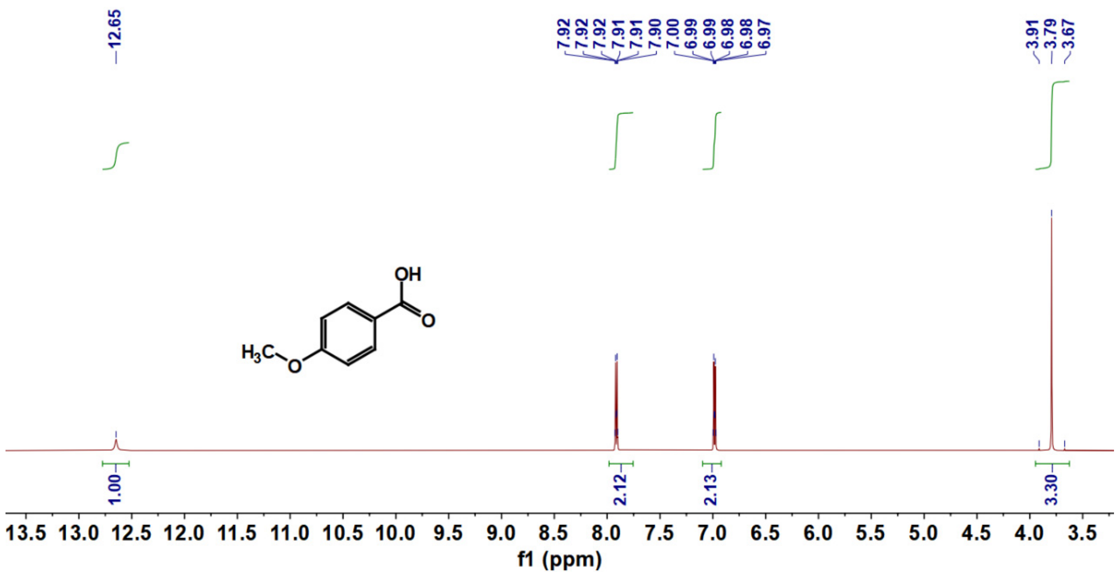

Supplementary Figure 34. ^{1}H NMR spectrum of the reaction product using 4-methylanisole as substrate in Figure 4a.

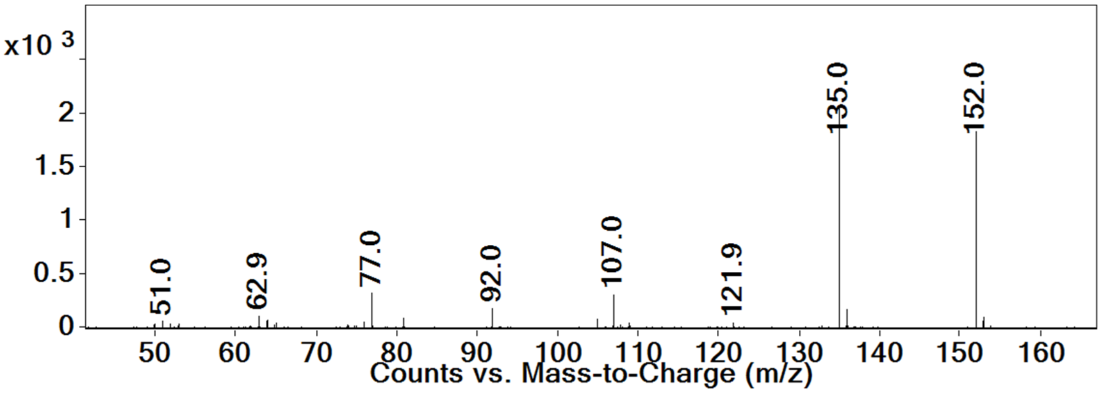

Supplementary Figure 35. Mass spectrum of the reaction product using 4-methylanisole as substrate.

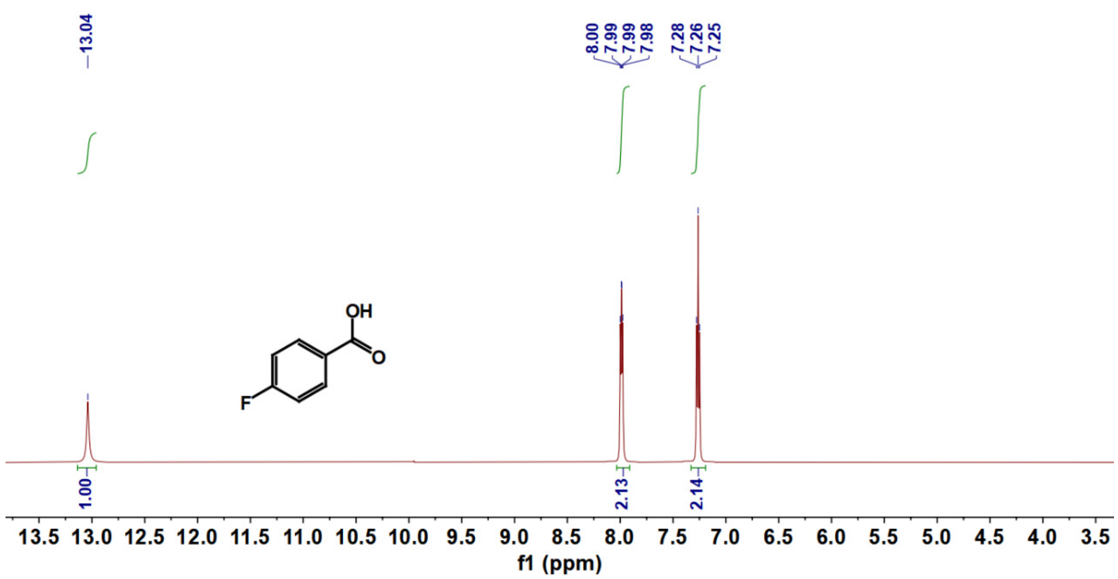

Supplementary Figure 36. ^{1}H NMR spectrum of the reaction product using p-fluorotoluene as substrate in Figure 4a.

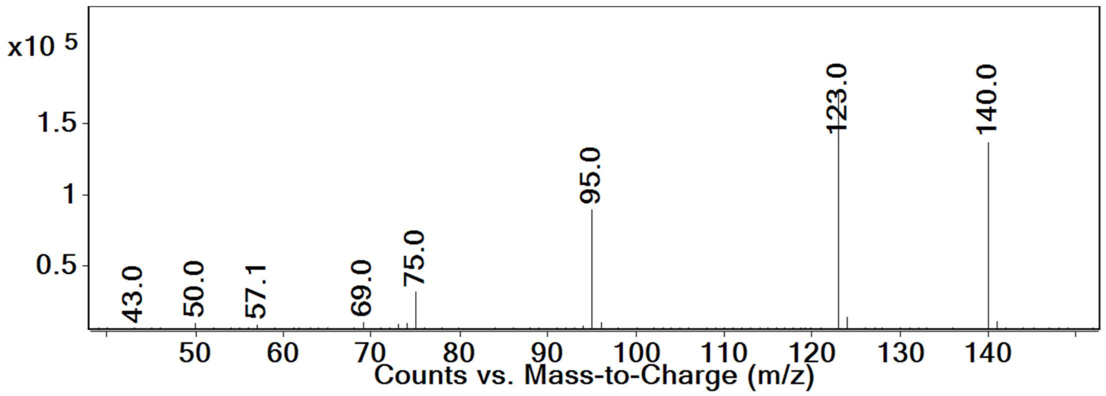

Supplementary Figure 37. Mass spectrum of the reaction product using p-fluorotoluene as substrate.

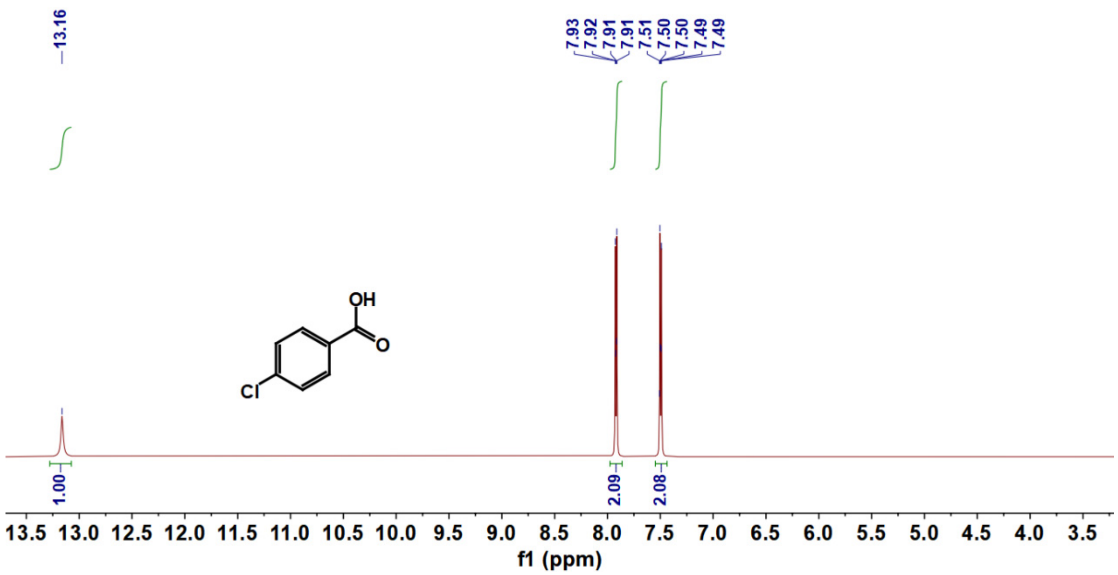

Supplementary Figure 38. ^{1}H NMR spectrum of the reaction product using 4-chlorotoluene as substrate in Figure 4a.

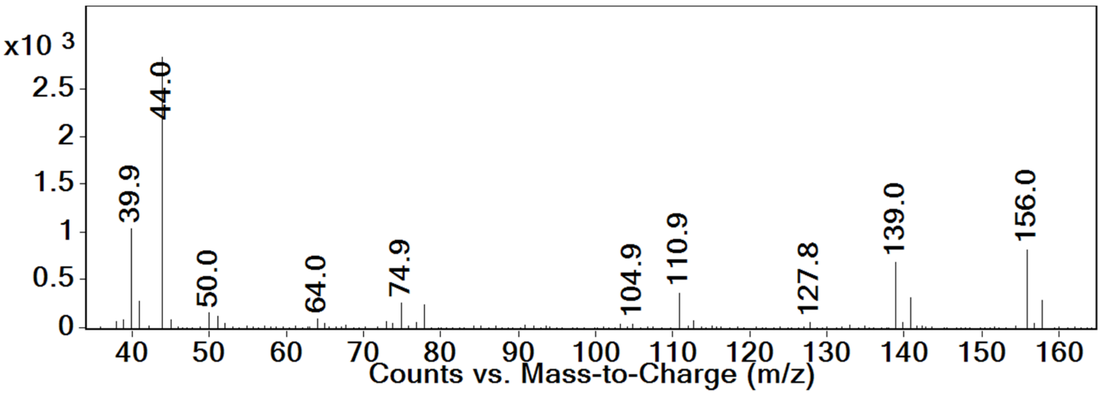

Supplementary Figure 39. Mass spectrum of the reaction product using 4-chlorotoluene as substrate.

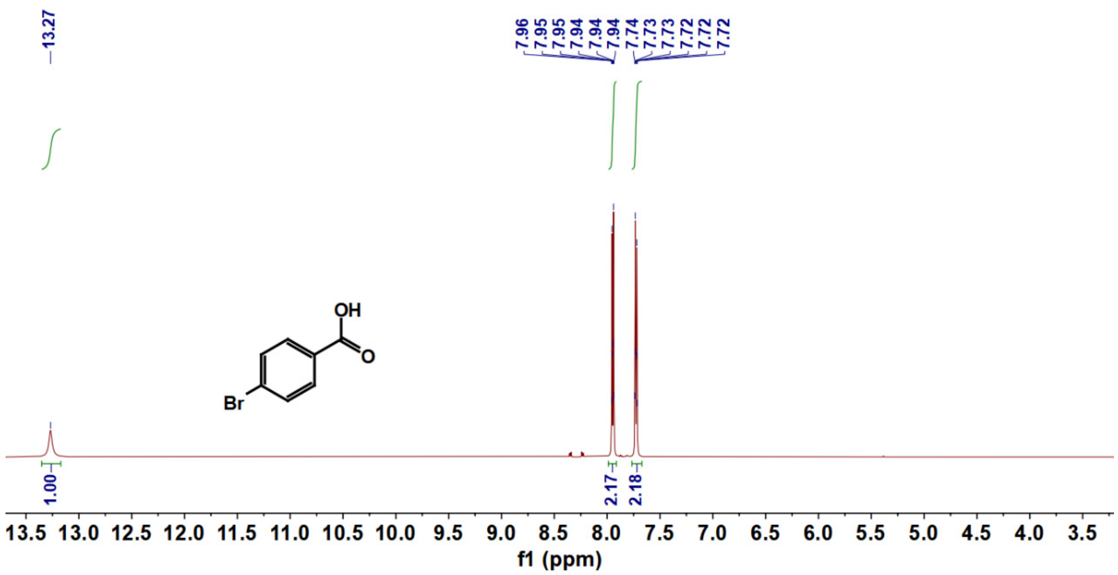

Supplementary Figure 40. ^{1}H NMR spectrum of the reaction product using p-bromotoluene as substrate in Figure 4a.

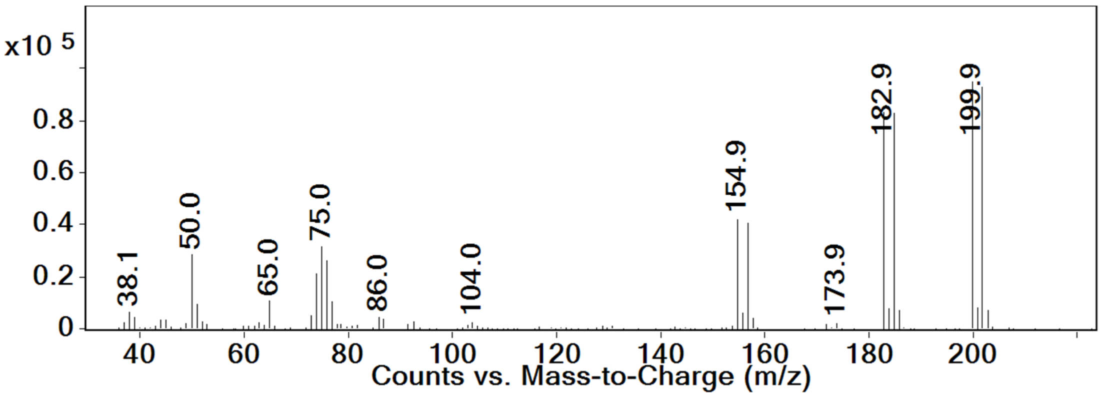

Supplementary Figure 41. Mass spectrum of the reaction product using p-bromotoluene as substrate.

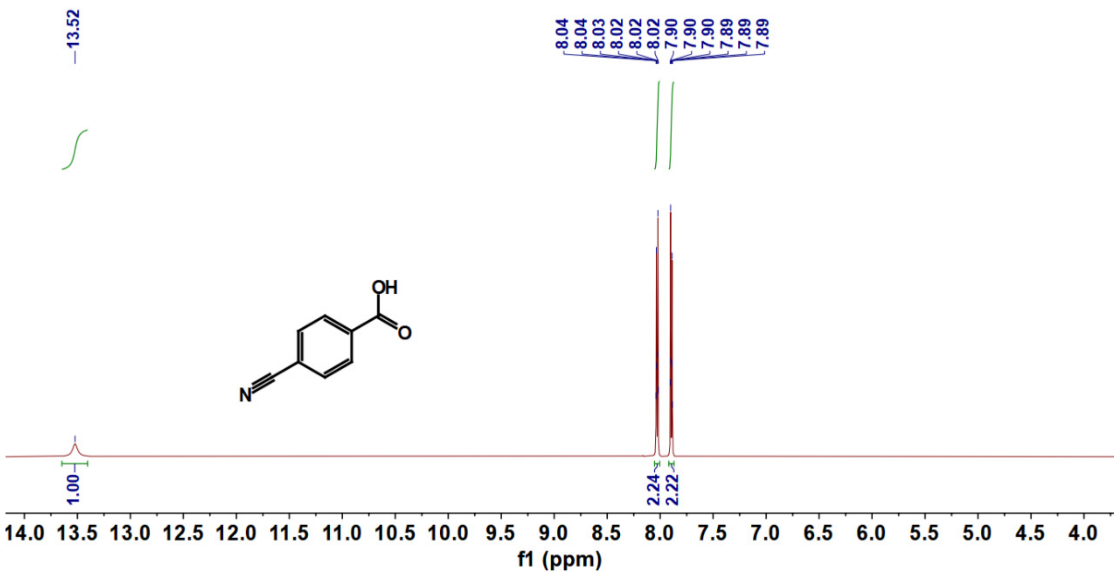

Supplementary Figure 42. ^{1}H NMR spectrum of the reaction product using p-tolunitrile as substrate in Figure 4a.

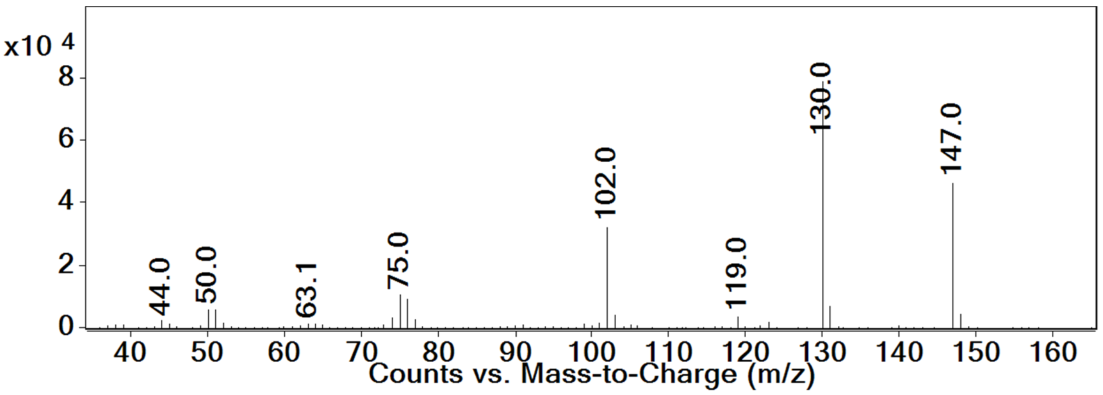

Supplementary Figure 43. Mass spectrum of the reaction product using p-tolunitrile as substrate.

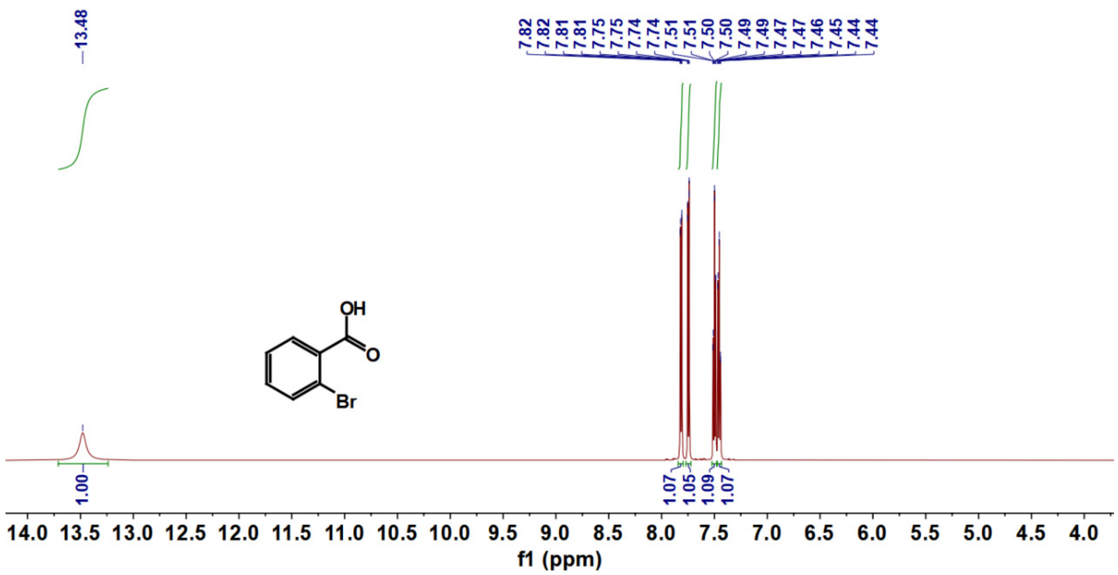

Supplementary Figure 44. ^{1}H NMR spectrum of the reaction product using 2-bromotoluene as substrate in Figure 4a.

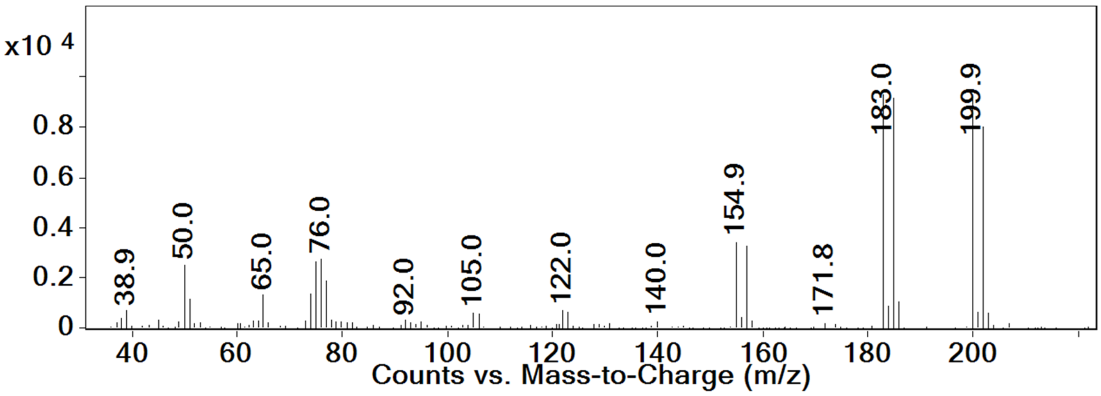

Supplementary Figure 45. Mass spectrum of the reaction product using 2-bromotoluene as substrate.

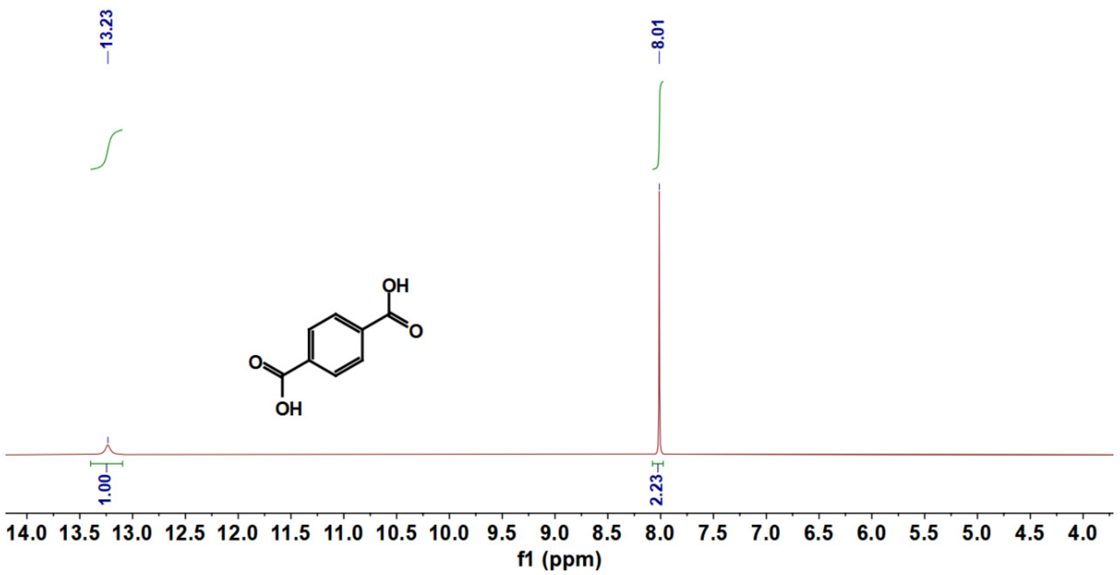

Supplementary Figure 46. ^{1}H NMR spectrum of the reaction product using paraxylene as substrate in Figure 4a.

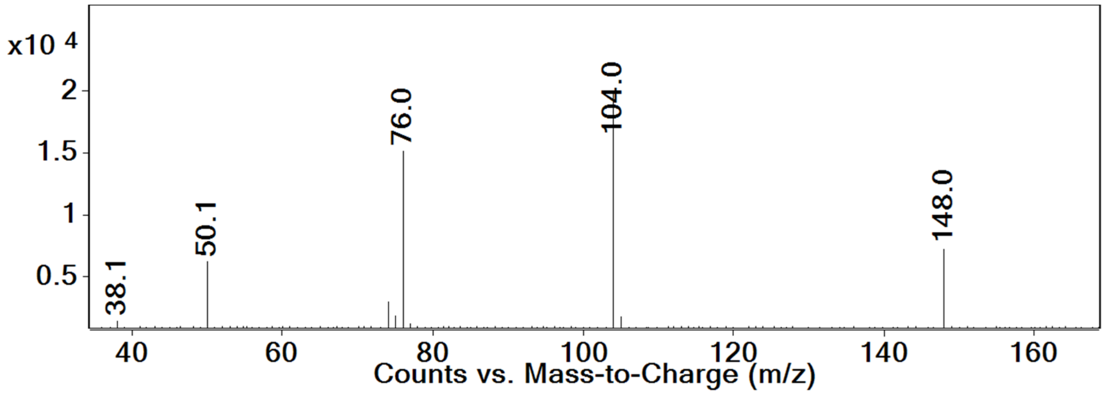

Supplementary Figure 47. Mass spectrum of the reaction product using paraxylene as substrate.

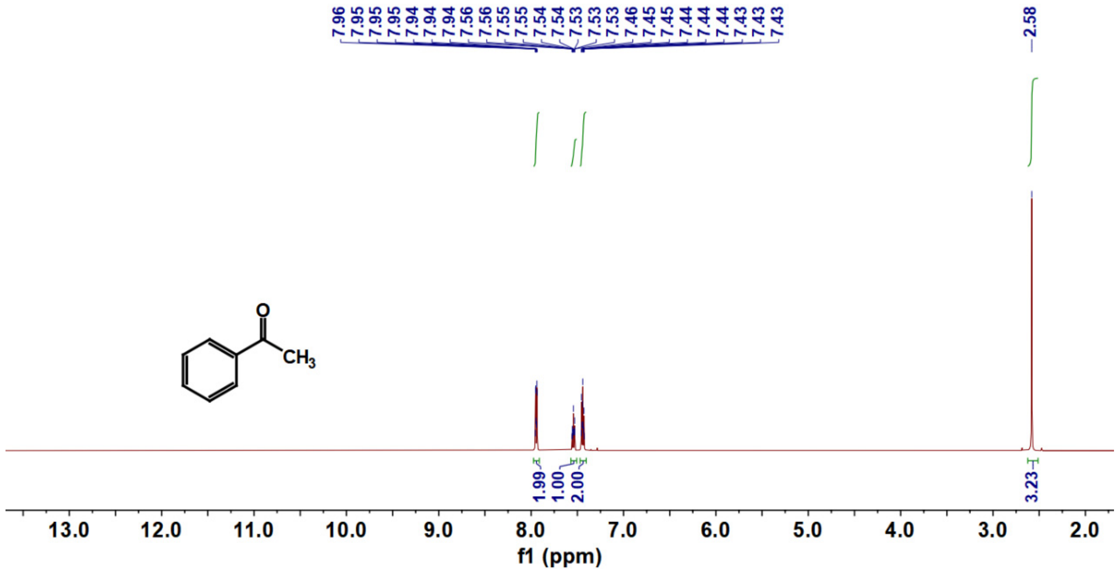

Supplementary Figure 48. ^1H NMR spectrum of the reaction product using ethylbenzene as substrate in Figure 4a.

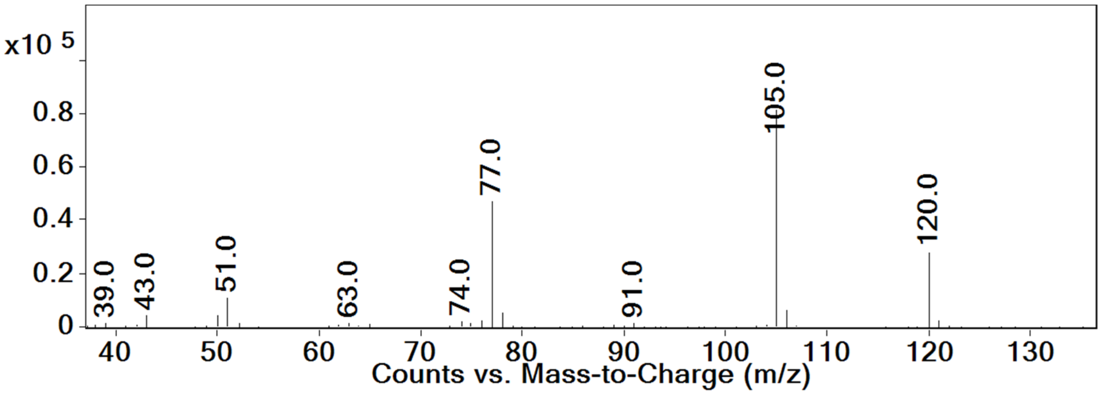

Supplementary Figure 49. Mass spectrum of the reaction product using ethylbenzene as substrate.

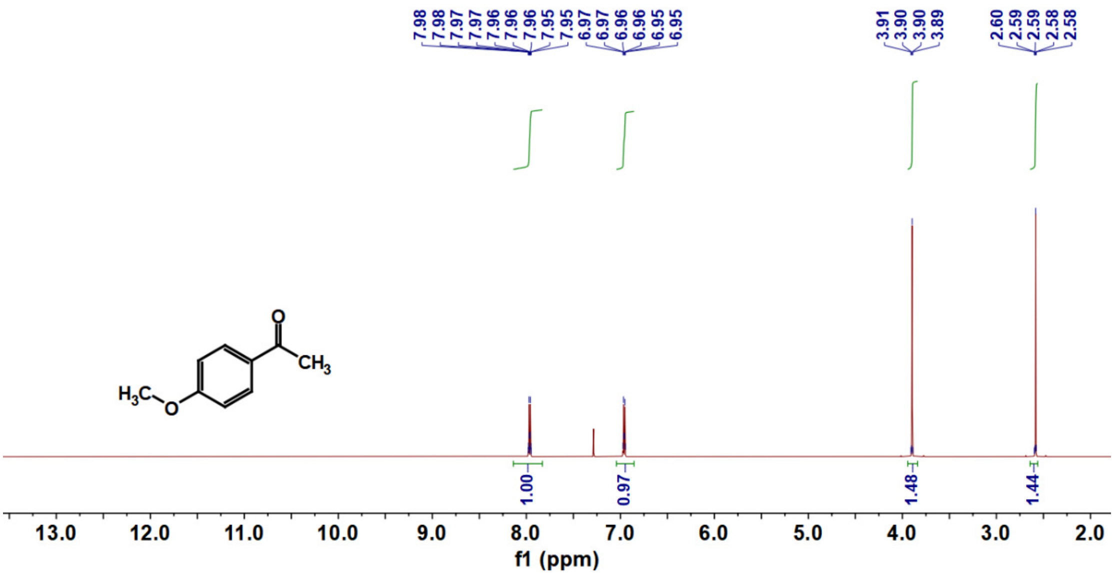

Supplementary Figure 50. ^{1}H NMR spectrum of the reaction product using 4-ethylanisole as substrate in Figure 4a.

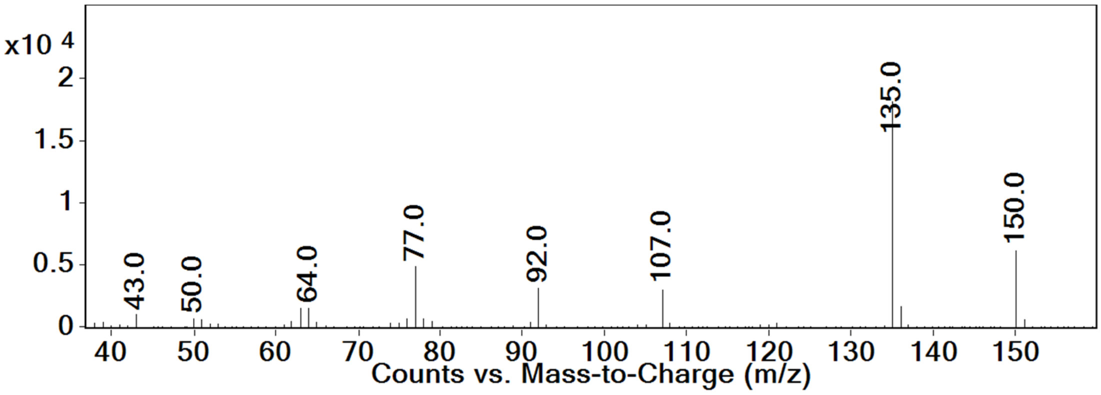

Supplementary Figure 51. Mass spectrum of the reaction product using 4-ethylanisole as substrate.

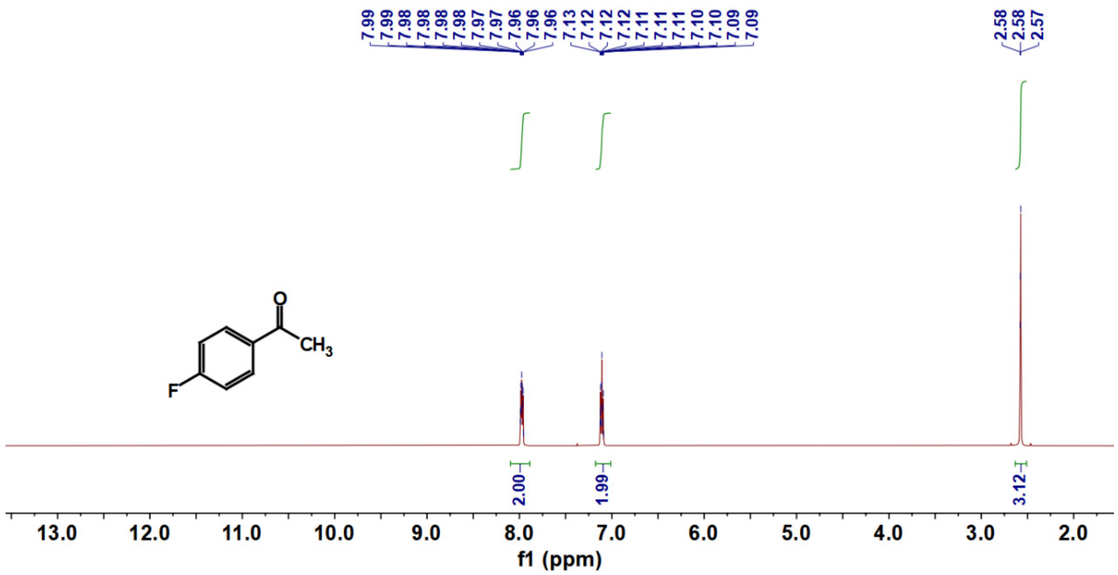

Supplementary Figure 52. ^{1}H NMR spectrum of the reaction product using 1-ethyl-4-fluorobenzene as substrate in Figure 4a.

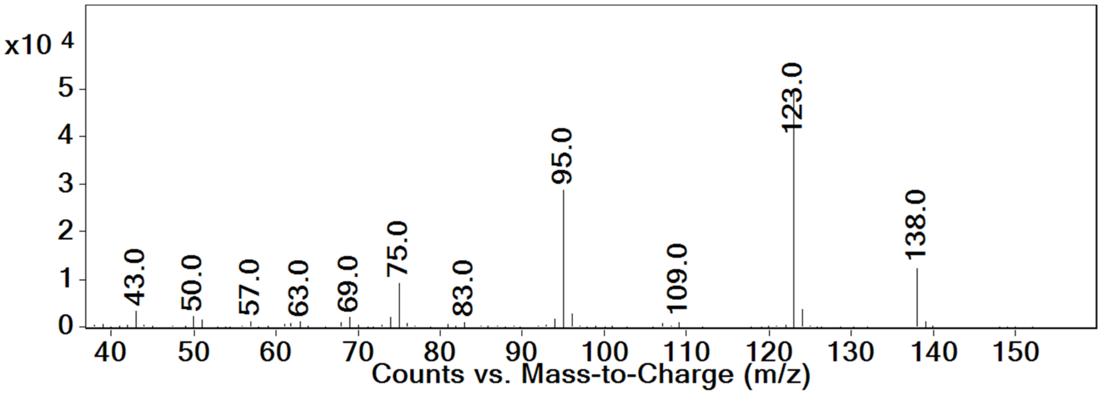

Supplementary Figure 53. Mass spectrum of the reaction product using 1-ethyl-4-fluorobenzene as substrate.

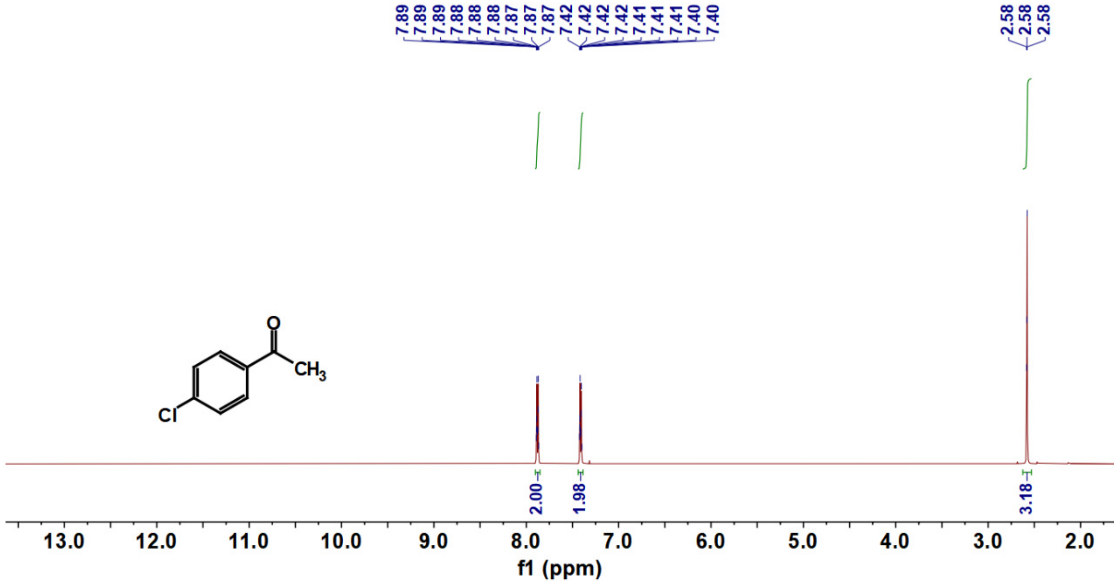

Supplementary Figure 54. ^{1}H NMR spectrum of the reaction product using 1-chloro-4-ethylbenzene as substrate in Figure 4a.

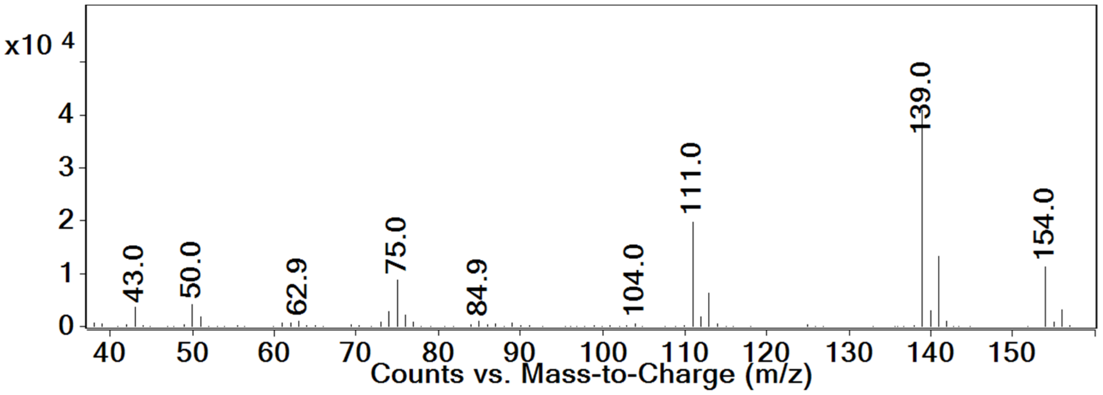

Supplementary Figure 55 Mass spectrum of the reaction product using 1-chloro-4-ethylbenzene as substrate.

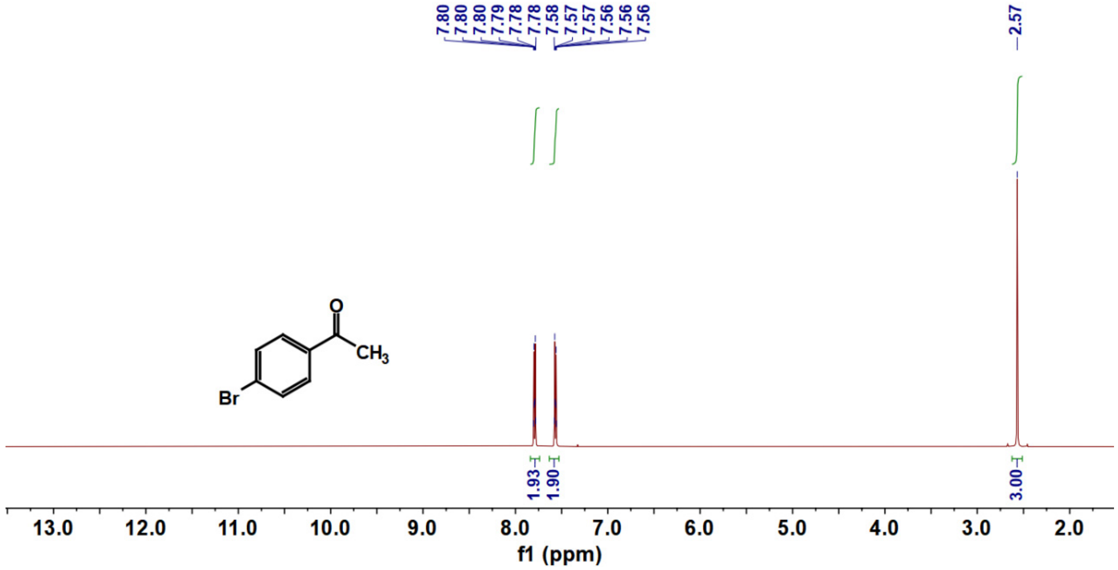

Supplementary Figure 56. ^{1}H NMR spectrum of the reaction product using 1-bromo-4-ethylbenzene as substrate in Figure 4a.

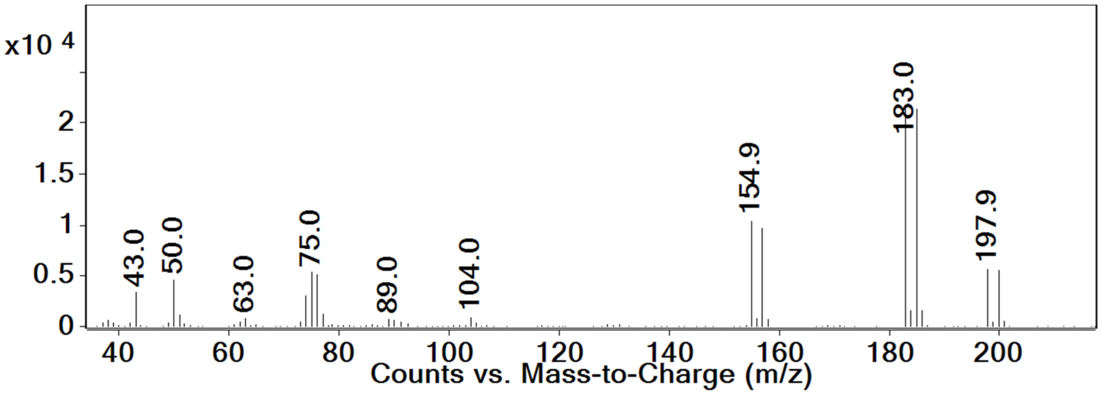

Supplementary Figure 57. Mass spectrum of the reaction product using 1-bromo-4-ethylbenzene as substrate.

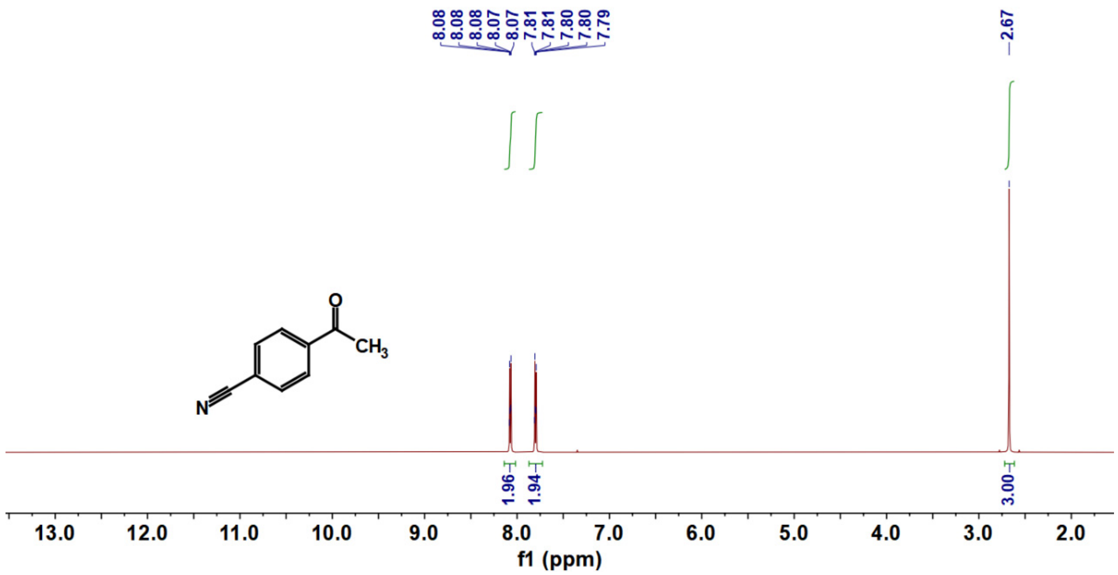

Supplementary Figure 58. ^{1}H NMR spectrum of the reaction product using 4-ethylbenzonitrile as substrate in Figure 4a.

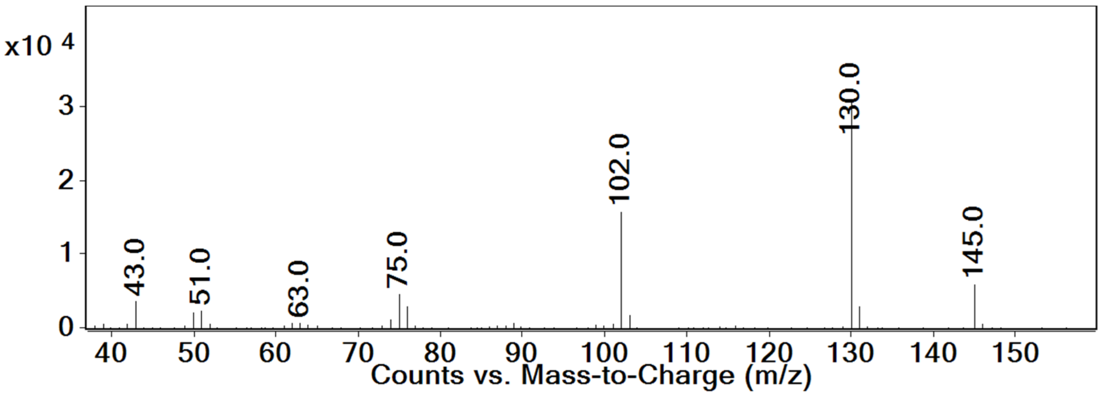

Supplementary Figure 59. Mass spectrum of the reaction product using 4-ethylbenzonitrile as substrate.

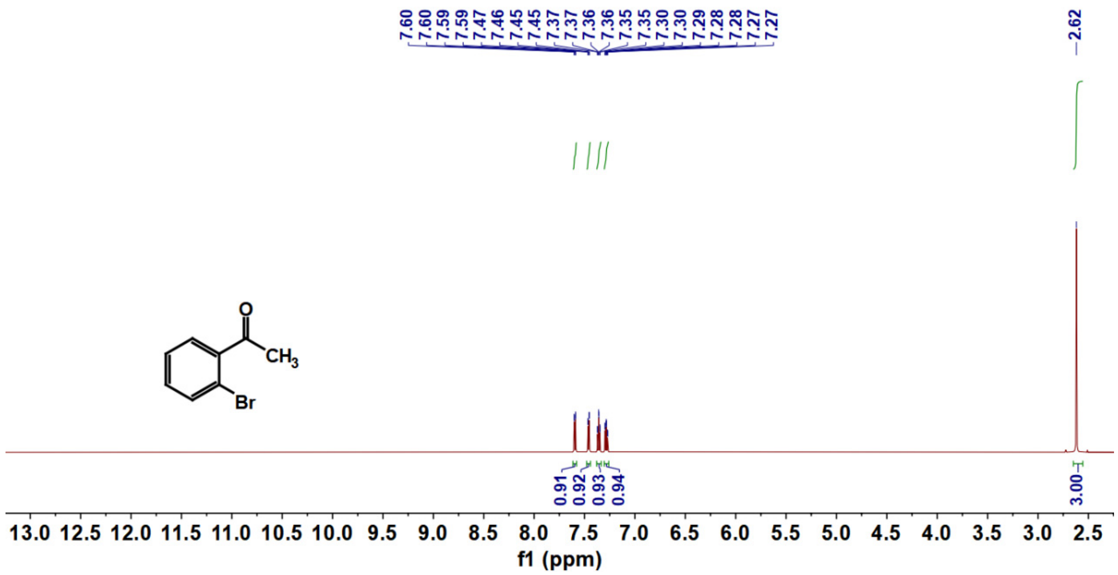

Supplementary Figure 60. ^{1}H NMR spectrum of the reaction product using 2-bromoethylbenzene as substrate in Figure 4a.

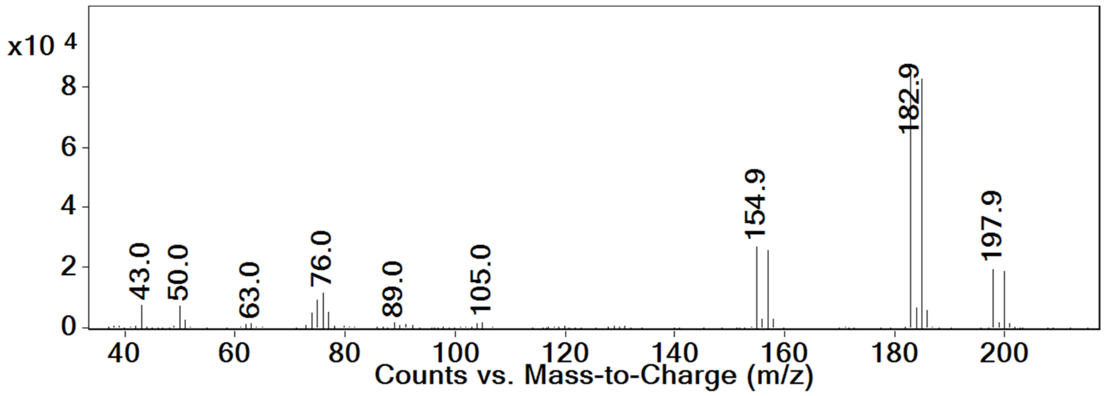

Supplementary Figure 61. Mass spectrum of the reaction product using 2-bromoethylbenzene as substrate.

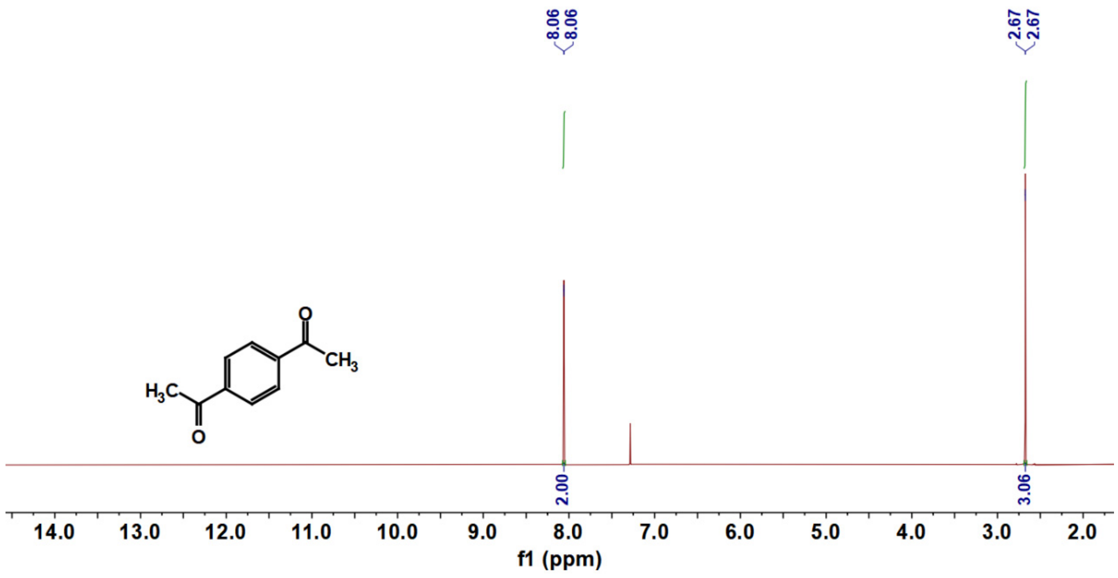

Supplementary Figure 62. ^{1}H NMR spectrum of the reaction product using 1,4-diethylbenzene as substrate in Figure 4a.

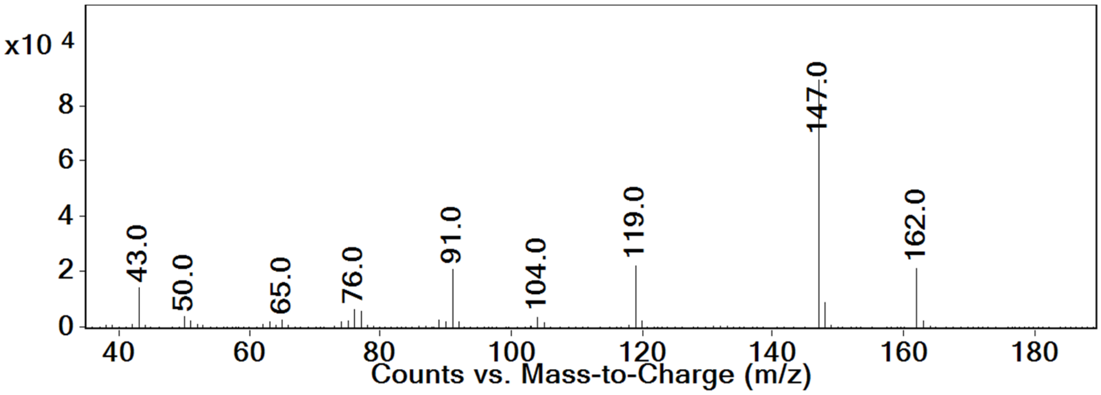

Supplementary Figure 63. Mass spectrum of the reaction product using 1,4-diethylbenzene as substrate.

Supplementary References

1. Bêche, E., *et al.* Ce *3d* XPS investigation of cerium oxides and mixed cerium oxide ($Ce_xTi_yO_z$). *Surf. Interface Anal.* **40**, 264-267 (2008).

2 Zhu, X. *et al.* Light and oxygen-enabled sodium trifluoromethanesulfinate-mediated selective oxidation of C-H bonds. *Green Chem.* **22**, 4357-4363 (2020).

3 Yuan, R. *et al.* Chlorine-Radical-Mediated Photocatalytic Activation of C-H Bonds with Visible Light. *Angew. Chem. Int. Ed.* **52**, 1035-1039 (2013).

4 Verma, S., Nasir Baig, R. B., Nadagouda, M. N. & Varma, R. S. Photocatalytic C-H Activation of Hydrocarbons over VO@g-C_3N_4. *ACS Sustainable Chem. Eng.* **4**, 2333-2336 (2016).

5 Mühldorf, B. & Wolf, R. C-H Photooxygenation of Alkyl Benzenes Catalyzed by Riboflavin Tetraacetate and a Non-Heme Iron Catalyst. *Angew. Chem. Int. Ed.* **55**, 427-430 (2016).

6 Dai, Y. *et al.* A Supported Bismuth Halide Perovskite Photocatalyst for Selective Aliphatic and Aromatic C-H Bond Activation. *Angew. Chem. Int. Ed.* **59**, 5788-5796 (2020).

7 Ma, S. *et al.* Redox-active and Brønsted basic dual sites for photocatalytic activation of benzylic C–H bonds based on pyridinium derivatives. *Green Chem.* **24**, 2492-2498 (2022).

8 Wu, J. *et al.* Brønsted acid-catalysed aerobic photo-oxygenation of benzylic C-H bonds. *Green Chem.* **25**, 940-945 (2023).

9 Cao, X. *et al.* Engineering Lattice Disorder on a Photocatalyst: Photochromic BiOBr Nanosheets Enhance Activation of Aromatic C-H Bonds via Water Oxidation. *J. Am. Chem. Soc.* **144**, 3386-3397 (2022).

10 Zhang, C. *et al.* Generation and Confinement of Long-Lived N-Oxyl Radical and Its Photocatalysis. *J. Am. Chem. Soc.* **140**, 2032-2035 (2018).

11 Li, J. *et al.* Cercosporin-bioinspired selective photooxidation reactions under mild conditions. *Green Chem.* **21**, 6073-6081 (2019).

12 Uygur, M. *et al.* Metal- and additive-free C-H oxygenation of alkylarenes by visible-light photoredox catalysis. *Green Chem.* **23**, 3392-3399 (2021).

13 Xiao, X. *et al.* A Unique Fe-N_4 Coordination System Enabling Transformation of Oxygen into Superoxide for Photocatalytic C-H Activation with High Efficiency and Selectivity. *Adv. Mater.* **34**, 2200612 (2022).

14 Liu, G. *et al.* A series of highly stable porphyrinic metal-organic frameworks based on iron–oxo chain clusters: design, synthesis and biomimetic catalysis. *J. Mater. Chem. A* **8**, 8376-8382 (2020).

15 Chen, H. *et al.* Selective Functionalization of Hydrocarbons Using a ppm Bioinspired Molecular Tweezer via Proton-Coupled Electron Transfer. *ACS Catal.* **11**, 6810-6815 (2021).

16 Tavallaei, H. *et al.* A Cooperative Effect in a Novel Bimetallic Mo–V Nanocomplex Catalyzed Selective Aerobic C-H Oxidation. *ACS Omega* **4**, 3601-3610 (2019).

17 Saha, R. & Sekar, G. Selective oxidation of alkylarenes to aromatic acids/ketone in water by using reusable binaphthyl stabilized Pt nanoparticles (Pt-BNP) as catalyst. *Appl. Catal. B* **250**, 325-336 (2019).

18 Wu, H. *et al.* Preparation of Copper Phosphate from Naturally Occurring Phytic Acid as an Advanced Catalyst for Oxidation of Aromatic Benzyl Compounds. *ACS Sustainable Chem. Eng.* **6**, 13670-13675 (2018).

19 Aguadero, A. *et al.* An Oxygen-Deficient Perovskite as Selective Catalyst in the Oxidation of Alkyl Benzenes. *Angew. Chem. Int. Ed.* **50**, 6557-6561 (2011).

20 Gao, Y. *et al.* Nitrogen-Doped sp^2-Hybridized Carbon as a Superior Catalyst for Selective Oxidation. *Angew. Chem. Int. Ed.* **52**, 2109-2113 (2013).

21 Kimberley, L. *et al.* The Origin of Catalytic Benzylic C-H Oxidation over a Redox-Active Metal-Organic Framework. *Angew. Chem. Int. Ed.* **60**, 15243-15247 (2021).

22 Zhou, J. *et al.* Chemoselective Oxyfunctionalization of Functionalized Benzylic Compounds with a Manganese Catalyst. *Angew. Chem. Int. Ed.* **61**, e202205983 (2022).

23 Sun, Y. *et al.* Nitrogen, Sulfur Co-doped Carbon Materials Derived from the Leaf, Stem and Root of Amaranth as Metal-free Catalysts for Selective Oxidation of Aromatic Hydrocarbons. *ChemCatChem* **11**, 1010-1016 (2019).

24 Yang, G., Ma, Y. & Xu, J. Biomimetic Catalytic System Driven by Electron Transfer for Selective Oxygenation of Hydrocarbon. *J. Am. Chem. Soc.* **126**, 10542-10543 (2004).

25 Liu, W. *et al.* Discriminating Catalytically Active FeNx Species of Atomically Dispersed Fe–N–C Catalyst for Selective Oxidation of the C-H Bond. *J. Am. Chem. Soc.* **139**, 10790-10798 (2017).

26 Liu, P. *et al.* ChemInform Abstract: Iron Oligopyridine Complexes as Efficient Catalysts for Practical Oxidation of Arenes, Alkanes, Tertiary Amines and N-Acyl Cyclic Amines with Oxone. *Chem. Sci.* **2**, 2187-2195 (2011).

27 Zhang, P. *et al.* Solvent-free aerobic oxidation of hydrocarbons and alcohols with Pd@N-doped carbon from glucose. *Nat. Commun.* **4**, 1593 (2013).

28 Yi, C. S., Kwon, K.-H. & Lee, D. W. Aqueous Phase C-H Bond Oxidation Reaction of Arylalkanes Catalyzed by a Water-Soluble Cationic Ru(III) Complex $[(pymox\text{-}Me_2)_2RuCl_2]^+BF^{4-}$. *Org. Lett.* **11**, 1567-1569 (2009).
